# Multimorbid life expectancy across race, socio-economic status, and sex in South Africa

**DOI:** 10.1080/00324728.2024.2331447

**Published:** 2024-05-16

**Authors:** Anastasia Lam, Katherine Keenan, Mikko Myrskylä, Hill Kulu

**Affiliations:** 1 University of St Andrews; 2 Max Planck Institute for Demographic Research; 3 University of Helsinki

**Keywords:** life expectancy, health expectancy, multimorbidity, South Africa, inequalities, intersectionality, cumulative advantage, race, sex, socio-economic status

## Abstract

Multimorbidity is increasing globally as populations age. However, it is unclear how long individuals live with multimorbidity and how it varies by social and economic factors. We investigate this in South Africa, whose apartheid history further complicates race, socio-economic, and sex inequalities. We introduce the term ‘multimorbid life expectancy’ (MMLE) to describe the years lived with multimorbidity. Using data from the South African National Income Dynamics Study (2008–17) and incidence-based multistate Markov modelling, we find that females experience higher MMLE than males (17.3 vs 9.8 years), and this disparity is consistent across all race and education groups. MMLE is highest among Asian/Indian people and the post-secondary educated relative to other groups and lowest among African people. These findings suggest there are associations between structural inequalities and MMLE, highlighting the need for health-system and educational policies to be implemented in a way proportional to each group’s level of need.

## Introduction

Multimorbidity is often defined as the co-occurrence of two or more chronic conditions (Johnston et al. 2019). As people accumulate multiple diseases, they are faced with decreased quality of life and increased healthcare requirements, which may include complex treatment regimens and multiple medications (Alaba and Chola 2013; Academy of Medical Sciences 2018). As populations age and the prevalence of non-communicable disease (NCD) risk factors (e.g. obesity) continues to rise globally, the burden of multimorbidity is expected to increase (Academy of Medical Sciences 2018). This has implications for individuals and health systems worldwide, but particularly in low- and middle-income countries (LMICs), which already face a large burden of NCD-related deaths (Basto-Abreu et al. 2022). Existing research on multimorbidity has been concentrated in high-income countries (HICs), but the literature on multimorbidity in LMICs has expanded in recent years (Abebe et al. 2020; Keetile et al. 2020; Khorrami et al. 2020; Roomaney et al. 2021; Kaluvu et al. 2022; Kamkuemah et al. 2022). Many LMICs are undergoing a protracted epidemiological transition, with likely overlaps in infectious and non-communicable disease stages, creating a dual burden of disease and different patterns of multimorbidity compared with those seen in HICs (Frenk et al. 1989; Oni et al. 2015). Multimorbidity often refers to NCDs, but this may not be the most representative definition in LMICs, because infectious diseases such as HIV and tuberculosis are also chronic (Farmer 2013).

In this paper, we focus on multimorbidity in South Africa, an upper-middle-income country with a complex history of inequalities and disease. Longitudinal evidence on multimorbidity in South Africa is lacking, and cross-sectional prevalence estimates vary widely, from 3 to 70 per cent, due to differences in age distributions and multimorbidity definitions across studies (Alaba and Chola 2013; Oni et al. 2015; Weimann et al. 2016; Chang et al. 2019; Roomaney et al. 2021). Across 10 studies of multimorbidity prevalence in South Africa, the five most common disease clusters included different combinations of hypertension, diabetes, HIV, and tuberculosis (Roomaney et al. 2021). This supports the idea that multimorbidity including both infectious diseases and NCDs is an important concern. Further, continuing high rates of infectious disease also contribute to the distribution of multimorbidity shifting to younger ages, since the average ages of people with HIV and tuberculosis are lower compared with those with only NCDs (Oni et al. 2014, 2015; Afshar et al. 2017). A recent study of people aged 15–24 with HIV in South Africa found that 5 per cent also had hypertension and 37 per cent had central obesity (Kamkuemah et al. 2022). Having these conditions at a young age increases the likelihood that multimorbidity will develop sooner. Additionally, antiretroviral therapy uptake has contributed to increases in life expectancy (LE) for people with HIV, leading to a higher chance of them developing multimorbidity earlier and also spending a greater share of their lives with multimorbidity (Oni et al. 2014, 2015; Doan et al. 2022).

LE has long been used as a measure of population health. However, as people began to live longer with non-fatal diseases, understanding how much time people were spending in states of good or bad health became more relevant. Development of an index of mortality and morbidity began in the 1960s and continued over the next few decades (Sanders 1964; Chiang 1965; Sullivan 1966, 1971; Ghana Health Assessment Project Team 1981; Bone 1992). This resulted in different measures of health expectancy, including disability-free LE, disability-adjusted LE, and healthy LE (Murray et al. 2000).

In this paper, we introduce the term ‘multimorbid life expectancy’ (MMLE): that is, the years someone is expected to live with multimorbidity. Previous studies have estimated MMLE but with different terminology, including ‘years lived with multimorbidity’ (Kingston et al. 2018; Chan et al. 2019), ‘time spent with multimorbidity’ (Chan et al. 2019), and ‘life years with multimorbidity’ (Tetzlaff et al. 2017; Botes et al. 2018). Other studies have not focused specifically on multimorbidity but on multiple chronic conditions, using such terms as ‘life expectancy with chronic morbidities’ (Payne 2022) and ‘duration of chronic disease’ (Murtaugh et al. 2011). The benefit of using a specific MMLE term is to encourage consistency, thus allowing for easier understanding and comparison of the multimorbidity burden between and within populations and over time, provided that the multimorbidity definitions are comparable.

We estimate LE and MMLE using an incidence-based Markov chain approach. This method makes maximum use of the first national household panel study in South Africa. It is currently the only approach that allows us to estimate the timings of transitions into and out of disease states accurately using panel data (Schneider et al. 2023). Using these timings, we can obtain expectancy estimates for various combinations of origin and destination states, as well as LE from any specified age.

Multimorbidity itself is already fraught with definitional complexity, and trying to understand it within the context of LE raises interesting questions. One major issue is whether longer time spent with multimorbidity might be considered a positive or negative outcome. On one hand, while disease accumulation might outwardly be thought of as a poor health outcome, longer survivorship with multiple conditions and a higher MMLE might reflect advantage through conditions that are less disabling and better disease management. On the other hand, lower MMLE might be a function of more severe conditions and poorer survivorship with those diseases.

Social and structural factors, such as race, socio-economic status (SES), and sex likely also play a role in determining an individual’s MMLE and how they experience it (Murtaugh et al. 2011; Chan et al. 2019; Payne 2022). In South Africa, apartheid was in place from 1948 to 1994 (Chopra and Sanders 2004), meaning that the majority of our study sample spent most of their lives under apartheid, with some being old enough to have experienced both pre- and post-apartheid society. This apartheid legacy has resulted in persistent health and social inequalities by sex and across racial and socio-economic strata (Chopra and Sanders 2004; Coovadia et al. 2009; Payne et al. 2020; Bell et al. 2022). Some individuals may experience more or fewer of these inequalities depending on their social location, and their advantages or disadvantages may be amplified over time by changing historical and political contexts. It is important to note that these social inequalities do not act independently: they interact with each other and likely have differential effects at various points in the life course, especially at times when major social and structural changes occur. Thus, we use intersectionality and cumulative advantage/disadvantage theory as lenses through which to view the numerous constellations of inequalities and try to understand how the varying pathways may influence multimorbidity and mortality.

Our study brings novelty to existing multimorbidity and LE research by using an incidence-based Markov chain approach with data from a middle-income country. Further, by incorporating theories of intersectionality and cumulative advantage/disadvantage, we conceptualize the complex interactions between race, SES, and sex that are deeply rooted in South African society. Using these approaches, we obtain a more holistic picture of the relationships between race, SES, sex, and MMLE in South Africa.

## Background

### Race

Race is a complex, socially constructed concept. It can contribute to differences in health and mortality outcomes, which may also vary depending on cultural, political, and country-specific factors. For example, in the United States, Black people consistently experience poorer health and higher rates of mortality compared with their white counterparts, whereas outcomes for Latinx and Asian people tend to be better (National Academies of Sciences 2017).

In South Africa, the systemic racial discrimination during apartheid had a detrimental impact on how different racial groups were treated. Racial classification provided the basis for the functioning of society, with individuals being declared as either white, Asian, Coloured, or Native (Posel 2001). Being ‘Coloured’ indicated that someone was of mixed race, and ‘Native’ was the label for Black South Africans. These racial categorizations remain ingrained in South African society, with younger generations (who did not directly experience apartheid) still self-identifying in one of these four groups. Based on these racial classifications, apartheid policies to improve health and the economy during most of the twentieth century focused on the white population, leaving the rest of the population with inferior healthcare and deteriorating living conditions (Benatar 2013). Black South Africans in particular had limited access to healthcare and it tended to be of poor quality; in 1980 the LE disparity between Black and white people was 15 years (Kon and Lackan 2008).

Although there have been improvements in health and social systems since 1994 (when apartheid ended), disparities remain entrenched and widespread. The health system evolved from highly fragmented region-specific systems during apartheid to a mixed and highly unequal pluralistic system post apartheid, in which only the wealthy could afford higher-quality private care (Kon and Lackan 2008). From 2012 to 2017, South Africa piloted the first phase of their National Health Insurance system as a step towards achieving universal health coverage and addressing the existing disparities; however, the results were mixed (Murphy and Moosa 2021). While coverage increased, the main issue was the lack of decentralized governance and, consequently, poor compliance in primary healthcare clinics and a lack of transparency and accountability (Day and Zondi 2019). The second implementation phase is currently in progress, with emphasis on legislation, including the National Health Insurance Bill which was passed in 2019 (Pauw 2022).

The direct association between multimorbidity and race is difficult to disentangle due to the inextricable link between race and SES that manifested under apartheid (Weimann et al. 2016). This may contribute to the lack of evidence on racial differences in multimorbidity in South Africa. Many multimorbidity studies have been conducted in rural areas, with almost all participants being Black (Oni et al. 2015; Wade et al. 2021; Wong et al. 2021), or have not included race in the analysis. Of the studies that have examined racial differences, two found that Asian/Indian participants were more than twice as likely to experience multimorbidity compared with African and Coloured participants (Weimann et al. 2016; Sewpaul et al. 2021). Another found that the odds of multimorbidity were about 1.5 times greater among Asian/Indian and Coloured participants compared with white participants (Phaswana-Mafuya et al. 2013).

### Socio-economic status

SES is often measured using an individual’s education, occupation, and/or income. Higher SES is often associated with better health and lower mortality (Mackenbach et al. 2008; Glymour et al. 2014; Lago et al. 2018). In LMICs, the patterns of associations between SES and certain health conditions or risk factors seem to differ from those seen in HICs. For example, there seems to be a positive association between SES and obesity in low-income countries, whereas the association in middle- and high-income countries is mixed (Dinsa et al. 2012). It has also been observed that in LMICs, low SES is associated with higher risk of certain cancers, cardiovascular disease, arthritis, and respiratory diseases, but there are mixed results for diabetes (Hosseinpoor et al. 2012; Sommer et al. 2015; Williams et al. 2018). Globally, lower SES is also associated with an increased risk of multimorbidity (Afshar et al. 2015; Arokiasamy et al. 2015; Pathirana and Jackson 2018). This relationship is also seen in South Africa, where socio-economically deprived individuals’ likelihood of multimorbidity is higher compared with those who are not deprived (Ataguba 2013; Weimann et al. 2016).

The evidence for an association between education and multimorbidity is mixed, with some studies reporting a positive association, some reporting a negative association, and some reporting no association (Feng et al. 2021). A cohort study from Brazil found that there may be a reduction in mortality risk for people with multimorbidity who are more highly educated, compared with those with less education (Bernardes et al. 2021). In South Africa, higher levels of education seem to be protective against multimorbidity (Alaba and Chola 2013; Afshar et al. 2015; Garin et al. 2016).

### Sex

Throughout this paper, we use the term ‘sex’ to describe people who are male or female, mainly because the questionnaires we used asked only about these binary categories. Additionally, the outcome of this study is related largely to physical and biological health differences. However, we want to acknowledge that many of the cultural, structural, and economic inequalities we might observe are more likely due to the social construction of gender. Thus, the instances in which we use men/women in the text are deliberate indications of gender.

Sex differences exist within health and mortality, with males tending to lead shorter but healthier lives compared with females. This phenomenon is known as the morbidity–mortality paradox or the male–female health-survival paradox. Males are generally more susceptible to fatal diseases earlier in life, whereas females experience more non-fatal diseases later in life (Rieker and Bird 2005). Females are also more likely to experience multimorbidity than males, and this pattern is consistent across various studies (Agur et al. 2016; Garin et al. 2016; Xu et al. 2017; Abebe et al. 2020). This may be due to females’ lower threshold for seeking treatment and their higher likelihood of attending primary care and being diagnosed with disease, but it could also be attributable to physiological factors, such as obesity or hormonal differences (Weimann et al. 2016; Afshar et al. 2017; Höhn et al. 2020). There are also social, cultural, and environmental factors that impact the health and mortality of males and females differently (Oksuzyan et al. 2010; Mateos et al. 2020).

The morbidity–mortality paradox is observed in South Africa, but the LE gap between females and males seems to be growing, in part due to differing rates of HIV-related mortality (Bor et al. 2015). Although women’s HIV prevalence is higher, men’s HIV-related mortality rates are higher, largely due to women being more likely to seek and complete treatment at earlier stages of the disease (Cornell et al. 2009, 2012; Kranzer et al. 2010; Bor et al. 2015; Haal et al. 2018). Regardless of HIV status, South African women exhibit higher rates of multimorbidity, hypertension, and obesity compared with men (Malaza et al. 2012; Oni et al. 2015; Chang et al. 2019; Wade et al. 2021; Wong et al. 2021).

### Intersectionality and cumulative advantage/disadvantage

The aforementioned factors of race, SES, and sex do not act independently on individuals, and their effects also vary by context. In combination with other characteristics such as age, sexuality, and disability, they are social stratifiers that interact and are shaped by the political, religious, cultural, and social environment (Hankivsky 2014). The term ‘intersectionality’ was first coined by Kimberlé Crenshaw when she wrote about how being Black and being a woman was a unique experience more complex than that of just being Black or just being a woman (Crenshaw 1989). Rather than being an additive approach, intersectionality describes the multidimensional interaction of various innate and acquired characteristics that shape human experiences (Hankivsky 2012; Bauer 2014). While it has been applied mainly as a way to explain the complexity of gender, race, and class through a feminist lens, it is gaining traction as a framework for understanding health disparities and the stigma associated with certain health conditions (Bowleg 2012, 2021; Bauer 2014; Jackson-Best and Edwards 2018; Turan et al. 2019).

Like intersectionality, cumulative advantage/disadvantage (CAD) theory describes how individuals’ lives are structured by the different risk and protective factors that surround them. The main tenet of CAD theory is the accumulation of these factors, resulting in some groups (e.g. less educated Black women) becoming progressively less advantaged while others (e.g. more highly educated white men) become more advantaged (Shuey and Willson 2008; Pais 2014). Over time, this advantage or disadvantage tends to grow, resulting in greater inequalities between the advantaged and disadvantaged groups (Diprete and Eirich 2006; Willson et al. 2007; Seabrook and Avison 2012; Dannefer 2020).

Using both intersectionality and CAD frameworks allows for a more nuanced interpretation of the mechanisms of inequality in MMLE. There has been limited research using these theories within the domain of multimorbidity. Rather, many studies have investigated mid- to later-life health more generally, often taking a life-course approach (Singh-Manoux 2004; Jackson and Engelman 2022). While CAD theory is not always explicitly mentioned, authors tend to use terms such as ‘cumulative socio-economic disadvantage’ or ‘accumulation of disadvantage’, which suggest similar ideas. Based on previous literature, we would expect individuals with more social and structural disadvantages to experience poorer health outcomes. However, in LMICs certain diseases and risk factors, such as diabetes and obesity, either tend to cluster in more advantaged groups or there is no clear pattern (Dinsa et al. 2012; Templin et al. 2019; Seiglie et al. 2020).

### Summary and hypotheses

Existing evidence indicates that race, SES, and sex display varying and potentially synergistic associations with multimorbidity. This study aims to quantify MMLE across race, SES, and sex groups. We use intersectionality and CAD theory as frameworks to interpret the complex relationships between these factors in South Africa.

Because of the strong correlation between race and SES, we anticipate that African people, whose SES tends to be lower, will spend more time with multimorbidity than white people, whose SES tends to be higher. Accordingly, we expect to see a reverse socio-economic gradient, with higher SES being associated with lower MMLE. We also expect that females will consistently exhibit higher MMLE than males, due to the morbidity–mortality paradox. Based on intersectionality theory, we foresee that the roles of race and SES will differ by sex, due to differences in gender norms across cultures and in access to education (Mabokela and Mawila 2004; Helman and Ratele 2016). From CAD theory, we expect groups with the most disadvantages (e.g. less educated African females) to experience higher MMLE compared with the least disadvantaged groups (e.g. more highly educated white males). However, there is also potential to find evidence for the opposite relationship, whereby MMLE is lower among the most disadvantaged groups and higher among the least disadvantaged groups.

## Methods

### Data

Data came from the National Income Dynamics Study (NIDS), a nationally representative household panel survey in South Africa, with five waves of data collected from 2008 to 2017 (Southern Africa Labour and Development Research Unit 2016a, 2016b, 2016c, 2018a, 2018b). The baseline sample was collected using a two-stage clustered sampling design and consisted of over 28,000 individuals from more than 7,300 households (Brophy et al. 2018). The survey collected a variety of data, including self-reported economic, socio-demographic, health/well-being, and household information.

We included participants who were interviewed using the adult (individual) questionnaire, since it was the only one that provided detailed health information, such as anthropometric measurements. Death was measured through household reports of deaths that had occurred in that household in the past 24 months. Merging data from all five waves for adults aged 20+ resulted in a total sample of *N*  =  28,237 participants. We excluded participants who were present at only one wave and subsequently lost to follow-up (*n*  =  9,340), those who did not respond to two or more adult questionnaires (*n*  =  447), and anyone missing essential socio-demographic or mortality information (*n*  =  420). The excluded participants were more likely to be male, slightly older, more highly educated, Asian/Indian or white, and living in urban areas than those in the analysis sample (Section I, supplementary material). This is in line with attrition patterns identified by the NIDS investigators, where 39 per cent of Asian/Indian and 52 per cent of white participants were not reinterviewed at the second wave (Branson 2019). Almost 11 per cent of the excluded participants were also missing disease information at baseline, because they were never successfully interviewed as individuals (their demographic information was obtained through household questionnaires).

The final analytic sample consisted of *n*  =  18,030 participants. Over half of participants were present in all five waves (51 per cent), 20 per cent were present in four, 13 per cent in three, and 16 per cent in only two waves. Each participant experienced anywhere from two to five transitions—remaining in the same state, transitioning to another disease state, or transitioning to death—depending on the number of waves in which they were present. We assume that the number of diseases they reported at Wave 1 was the same number they had before participating in the survey, which counted as one transition; this resulted in a total of *n*  =  73,248 transitions.

### Variables

#### Measurement of multimorbidity

There is no consensus on a multimorbidity definition. While it is usually defined as the co-occurrence of two or more chronic conditions, the number and type of included diseases varies substantially (Academy of Medical Sciences 2018; Johnston et al. 2019; Ho et al. 2021). There are several different methods of measuring multimorbidity, including simple disease counts, weighted disease indices, and weighted medication indices (Ho et al. 2021). The range of diseases included in multimorbidity can also range from two to over 100 and relates partly to whether disease data are obtained through self-report or medical records (Ho et al. 2021). Additionally, the inclusion of certain diseases, such as HIV and tuberculosis, seems to be context dependent (Ho et al. 2021). Unless focusing specifically on NCDs, South African studies on multimorbidity have often included HIV and tuberculosis because they are both endemic chronic infections (Alaba and Chola 2013; Ataguba 2013; Weimann et al. 2016; Peltzer 2018; Chang et al. 2019; Sharman and Bachmann 2019; Roomaney et al. 2021; Wade et al. 2021; Wong et al. 2021; Roomaney et al. 2022).

Our study included a total of 14 chronic diseases: Alzheimer’s disease, arthritis, asthma, cancer, diabetes, emphysema, epilepsy, heart problems, HIV, hypertension, kidney problems, psychological/psychiatric disorders, stroke, and tuberculosis. Participants were asked explicitly if a doctor, nurse, or healthcare professional had ever told them they had asthma, cancer, diabetes, heart problems, stroke, or tuberculosis. Hypertension was defined based on the average measures of systolic and diastolic blood pressure, which were taken twice at each wave. The cut-offs for hypertension were recording a systolic blood pressure ≥140 mmHg and a diastolic blood pressure ≥90 mmHg, with the difference between the two being ≥15 mmHg (Cois and Ehrlich 2018; Unger et al. 2020). Alzheimer’s disease, arthritis, emphysema, epilepsy, HIV, kidney problems, and psychological/psychiatric disorders were obtained through responses to the question, *Do you have any other major illness or disability not mentioned above?* Most of these diseases were provided as optional answers to the question, except for arthritis and kidney problems, which were obtained by searching answers from the free response ‘Other’ option.

We defined multimorbidity as closely as possible to Ho et al.’s (2021) list of 20 core chronic conditions that should be included in measures of multimorbidity; their list was derived after a review of over 566 multimorbidity studies. See Section II, supplementary material, for a diagram of how our included diseases overlap with Ho et al.’s (2021) core list. Hypertension and epilepsy were not included in Ho et al.’s (2021) core list but were included in about 70 and 20 per cent of their reviewed studies, respectively (Ho et al. 2021). Additionally, HIV and tuberculosis were not in their core list of 20, but it was noted that they could be considered core conditions in certain contexts, such as South Africa. While we did not specifically include the core conditions of coronary heart disease and heart failure, these are likely captured within our ‘heart problems’ group. Similarly, depression, schizophrenia, anxiety, and potentially alcohol and drug problems are likely captured by our ‘psychological/psychiatric disorders’ group. Additionally, although we did not have a measure of chronic obstructive pulmonary disease (COPD), we included emphysema, which is a type of COPD. Core conditions mentioned in response to the question on other major illness that we did not include in our definition are chronic liver disease and chronic pain; these were excluded because each response was given by under 10 people. Vision problems and musculoskeletal impairment due to injury are also on Ho et al.’s (2021) core list, but due to the nature of the survey questions, we could not untangle vision problems from the overall ‘problems with sight, hearing, or speech’ category and could not attribute any reported musculoskeletal impairment or physical disability to an injury.

#### Other covariates

Age was included as a continuous variable, starting from 20 years, and no upper limit restrictions were used. Residence was defined as living in either an urban or rural area, with ‘urban’ indicating any built-up areas and ‘rural’ indicating villages under jurisdiction of traditional leaders and also farms (Brophy et al. 2018). Participants were asked to identify as being part of one of the following racial groups: African, Asian/Indian, Coloured, or white. Here, ‘African’ is used to describe those of the native Black African group, and ‘Coloured’ is a uniquely southern African term used to describe someone of mixed-race ancestry (Adhikari 2009), as used in both the NIDS survey and South Africa’s census. Education was measured as the highest level of completed education at the baseline visit and split into three categories: less than secondary school (Grade 8 or below), secondary school (Grades 9–12), and post-secondary education (including vocational training). We chose education as our SES indicator because it is commonly used in health research, being easy to measure and generally staying consistent throughout life (Galobardes 2006).

### Statistical analysis

**Figure 1:**
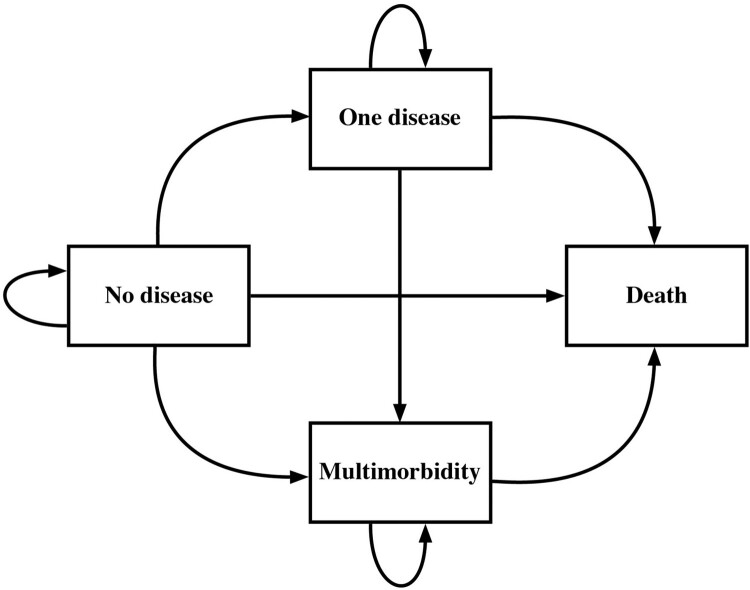
State space of the Markov model *Source*: Authors’ own.

We obtained descriptive statistics of our analytic sample and computed the prevalence of each of the included diseases to identify the most common diseases and multimorbid combinations. We then used multinomial logit models to predict the probability of transitioning between four different states: no disease, one disease, multimorbidity, and death. Figure 1 shows that individuals can begin in any of the three disease states and either remain in the same state, transition to a subsequent state, or die. We did not allow for reverse transitions, in which individuals might become cured, due to the chronic nature of the diseases. Death is an absorbing state, meaning that once someone enters that state they cannot leave. All other states are considered transient.

The base model (Model 1) was adjusted for linear age and residence, stratified by sex, and weighted using NIDS design weights, which correct for non-response. Details of how the weights were calculated can be found elsewhere (Brophy et al. 2018). Building on Model 1, we ran the subsequent models with the addition of the following variables: race (Model 2), education (Model 3), or the interaction between race and education (Model 4). The models take the general form:

(1) $$\log\left(\frac{\;p_{\mathrm{ij}}}{\;p_{\mathrm{iN}}}\right)=\alpha_{\mathrm{ij}}+\beta_{1,\mathrm{ij}}\mathrm{Age}+\beta_{2,\mathrm{ij}}\mathrm{Res}+\gamma_{\mathrm{ij}}\mathrm{Cov},\;$$

where $p_{\mathrm{ij}}$ is the biennial probability of moving from state *i* to state *j*; *j = N* indicates the reference destination state of no disease; $\alpha_{\mathrm{ij}}$ is the intercept; *Age* is the individual’s exact age at each interview; *Res* indicates the baseline proportion of participants living in urban areas; and $\gamma_{\mathrm{ij}}$ is the coefficient for *Cov,* which includes the covariates race, education, or their interaction. The race and education variables in Model 4 were simplified due to small sample sizes in some of the original strata: the Asian/Indian racial group was excluded, and education was dichotomized into ‘less than secondary school’ and ‘secondary school or more’ to create a balanced distribution across the two categories.

The transition probabilities were estimated by setting the residence variable to its sample proportion (i.e. proportion in urban areas) and setting the categorical variables to equal a specific group (e.g. African or post-secondary educated). These probabilities were input into a matrix with each row representing two-year age intervals from ages 20 to 85. The transition probability matrix was truncated at age 85 because of small sample sizes at the oldest ages. Each column of the matrix represents the probability of transitioning from state *i* to state *j*.

The predicted transition probabilities were subsequently input into discrete-time multistate Markov models. They take a Markov assumption, meaning that expectancy estimates are calculated based on the current state and covariate profile of an individual, regardless of their status or duration spent in any prior state (Kemeny and Snell 1983). They are analysed using a matrix notation, with transition probabilities organized into matrices *P = [p_ij_]* for each stratum of race and education, separately for males and females, by two-year age intervals (Hale et al. 2020; Lorenti et al. 2020). Equation (2) shows a simplified transition matrix:

(2) $$P_{\mathrm{ij}}=\left(\begin{array}{cccc} \;p_{11} & \;p_{12} & \;p_{13} & \;p_{14}\\ 0 & \;p_{22} & \;p_{23} & \;p_{24}\\ 0 & 0 & \;p_{33} & \;p_{34} \end{array}\right),\;$$

where $p_{11}$ represents the probability of remaining in the first state (no disease), $p_{12}$ the probability of moving from no disease to one disease, $p_{13}$ the probability of transitioning from no disease to multimorbidity, $p_{14}$ the probability of transition from no disease to death, and so on. The zeroes demonstrate that reverse transitions cannot occur (e.g. the transition from multimorbidity to one disease). Each transition occurs between an age interval *k* to *k + 1* (e.g. age interval 20–22 to 22–24), but this notation is excluded from the matrix in Equation (2) for simplicity.

These transition matrices were used to compute the time spent in each transient state through the fundamental matrix:

(3) $$N=(I_{n}-U{)}^{-1},\;\;$$

where U is a transient-state-only transition matrix; $I_{n}$ is an n×n identity matrix; and superscript −1 denotes the inverse. The row sums of N indicate the LE given origin state i. The expected time in destination state *j,* given an origin state *i,* is represented by $N_{\mathrm{ij}}$ (Hale et al. 2020; Lorenti et al. 2020).

We calculated truncated life expectancies from each origin state at age 20 until age 85 using the standard approach. This involved calculating expectancies conditional on our initial age of 20 and then obtaining a weighted average across the conditional expectancies. The weights corresponded to the origin state distribution of individuals at the starting age, but we calculated these for participants aged 20–29 to increase sample size and also obtained these distributions separately for males and females. We present only the weighted expectancies, but expectancies from each origin state (no disease, one disease, and multimorbidity) can be found in Section III (supplementary material). We also present MMLE in terms of absolute years and proportion of remaining LE. The 95 per cent confidence intervals are based on asymptotic theory and the delta method. This method does not set restrictions on the confidence limits, thus allowing negative confidence limits to occur. In cases where this occurred, the limit was set equal to zero, as negative expectancies are not possible. The calculations for these 95 per cent confidence intervals have recently been developed by Schneider (2023b). The underlying variance–covariance matrix of the multinomial logit model accounts for the complex survey design.

We performed sensitivity analyses for different definitions of multimorbidity and also for state and life expectancies from age 40 (rather than age 20). Hypertension is often referred to as a risk factor for disease but is also commonly included in multimorbidity studies (Stanaway et al. 2018; The Lancet 2019; Ho et al. 2021). Tuberculosis is often described as a chronic infectious disease, but many studies have investigated multimorbidity in people with tuberculosis rather than including it as one of the multimorbid diseases (Reis-Santos et al. 2013; Peltzer 2018; Siddiqi et al. 2020; Stubbs et al. 2021). Thus, we excluded hypertension and/or tuberculosis from our multimorbidity definition to see what impact it had on the expectancy estimates. We estimated LE at age 40 because we were interested in whether the patterns of time with multimorbidity and LE would change given an older initial age, since multimorbidity prevalence increases with age.

Analyses were conducted in both R version 4.0.3 (R Core Team 2020) and Stata 17 (StataCorp 2021) to ensure robustness across software. Expectancy estimates were obtained using the *mcwr* package in R (Schneider et al. 2021) and expectancy estimates with confidence intervals were obtained using the *dtms* package in Stata (Schneider 2023a).

## Results

Table 1 details the socio-demographic characteristics of our sample. The average age is 42.0 years (standard deviation (SD) 16.2), with males being younger than females. Ages range from 20 to 106 years, and 60 per cent of the sample is female. Most participants are African (80 per cent), followed by the Coloured (15 per cent), white (4 per cent), and Asian/Indian (1 per cent) groups. Almost half the participants (46 per cent) have received less than secondary school education, while 42 per cent have finished or completed at least some secondary school and 12 per cent have post-secondary education.

**Table 1:** Baseline socio-demographic characteristics and distribution of transitions between states of the analytic sample, by sex and overall: South Africa, 2008–17

	Male (*n* = 7,224)	Female (*n* = 10,806)	Overall (*n* = 18,030)
*Age (years)*			
Mean (SD)	40.9 (15.6)	42.7 (16.5)	42.0 (16.2)
*Race*			
African (%)	5,633 (78.0)	8,777 (81.2)	14,410 (79.9)
Coloured (%)	1,158 (16.0)	1,508 (14.0)	2,666 (14.8)
White (%)	336 (4.7)	393 (3.6)	729 (4.0)
Asian/Indian (%)	97 (1.3)	128 (1.2)	225 (1.2)
*Education level*			
Less than secondary school (%)	3,149 (43.6)	5,181 (47.9)	8,330 (46.2)
Secondary school (%)	3,140 (43.5)	4,360 (40.3)	7,500 (41.6)
Post-secondary education (%)	935 (12.9)	1,265 (11.7)	2,200 (12.2)
*Residence*			
Rural (%)	3,393 (47.0)	5,591 (51.7)	8,984 (49.8)
Urban (%)	3,831 (53.0)	5,215 (48.3)	9,046 (50.2)
*Origin ‘from’ state*			
No disease (%)	5,509 (76.3)	7,062 (65.4)	12,571 (69.7)
One disease (%)	1,319 (18.3)	2,707 (25.0)	4,026 (22.3)
Multimorbidity (%)	396 (5.5)	1,037 (9.6)	1,433 (8.0)
*Total number of transitions*			
No disease → No disease (%)	17,300 (88.5)	21,800 (87.3)	39,100 (87.8)
No disease → One disease (%)	1,599 (8.2)	2,405 (9.6)	4,004 (9.0)
No disease → Multimorbidity (%)	221 (1.1)	394 (1.6)	615 (1.4)
No disease → Dead (%)	438 (2.2)	381 (1.5)	819 (1.8)
One disease → One disease (%)	5,351 (83.1)	10,781 (84.5)	16,132 (84.0)
One disease → Multimorbidity (%)	727 (11.3)	1,559 (12.2)	2,286 (11.9)
One disease → Dead (%)	362 (5.6)	415 (3.3)	777 (4.0)
Multimorbidity → Multimorbidity (%)	2,358 (88.8)	6,424 (93.7)	8,782 (92.3)
Multimorbidity → Dead (%)	298 (11.2)	435 (6.3)	733 (7.7)

*Note:* For transitions, percentages sum to 100 within each origin state.

*Source*: Authors’ analysis of data from South African National Income Dynamics Study (2008–17).

**Figure 2:**
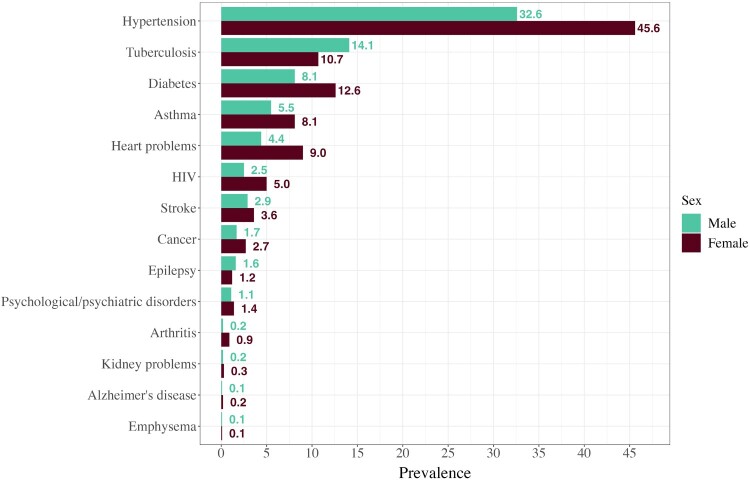
Baseline prevalence (in percentages) of each disease included in our definition of multimorbidity: males and females, South Africa, 2008–17 *Source*: Authors’ analysis of data from South African National Income Dynamics Study (2008–17).

More males than females entered the study without any of the included diseases (76 vs 65 per cent, respectively), whereas more females entered with one disease (25 vs 18 per cent) or multimorbidity (10 vs 6 per cent). Figure 2 shows the most prevalent diseases at baseline, the top three of which are hypertension (females 46 per cent; males 33 per cent), tuberculosis (females 11 per cent; males 14 per cent), and diabetes (females 13 per cent; males 8 per cent). In terms of multimorbidity (not shown), for males the three most common multimorbid clusters are hypertension & diabetes (17 per cent), hypertension & tuberculosis (10 per cent), and hypertension & heart problems (9 per cent). For females, the three most common multimorbid clusters are hypertension & diabetes (20 per cent), hypertension & heart problems (8 per cent), and hypertension & asthma (6 per cent).

### Transitions and transition probabilities

**Figure 3:**
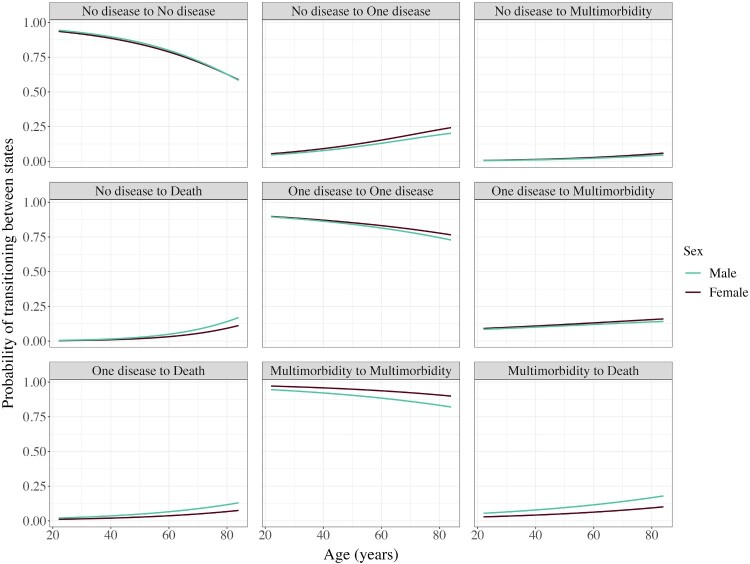
Probabilities of remaining in the same state or transitioning to a subsequent state by age: males and females, South Africa, 2008–17 *Source*: As for Figure 2.

Table 1 and Figure 3 show that most people remain in the state they were in when they entered the study (83–94 per cent depending on sex and origin state). The most common transition is from one disease to multimorbidity for both males (11 per cent of those with one disease initially) and females (12 per cent). Figure 3 also shows how transition probabilities change with age. The probabilities of remaining in the same state decrease with age, while probabilities of transitioning to a subsequent disease state or death increase.

**Figure A1:**
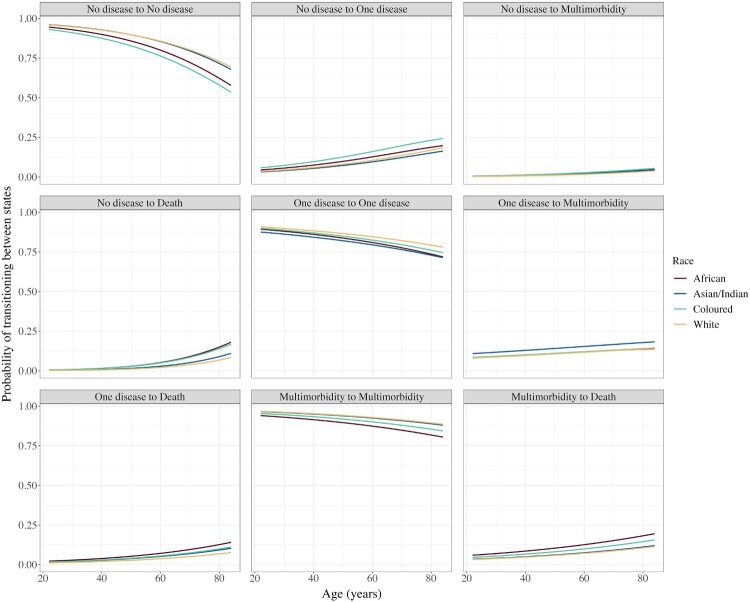
Males: probabilities of remaining in the same origin disease state or transitioning to a subsequent disease state or death over time, by race *Source*: Authors’ analysis of data from South African National Income Dynamics Study (2008–17).

**Figure A2:**
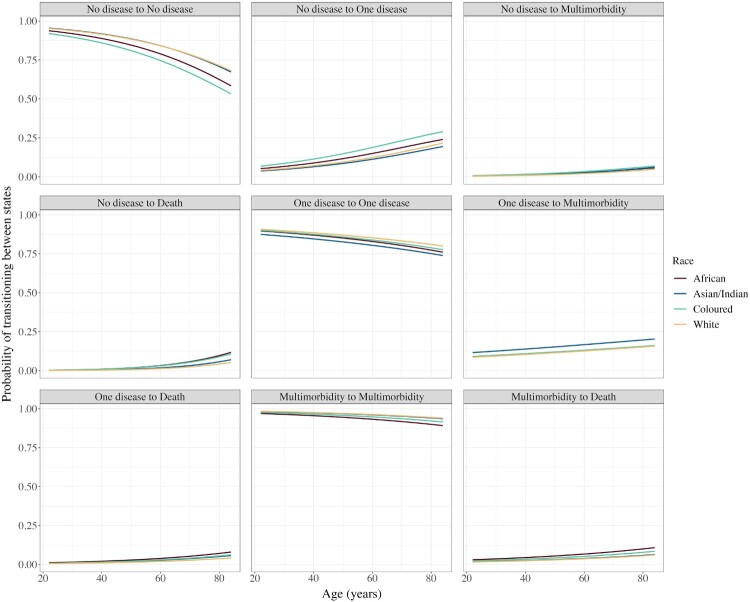
Females: probabilities of remaining in the same origin disease state or transitioning to a subsequent disease state or death over time, by race *Source*: As for Figure A1.

**Figure A3:**
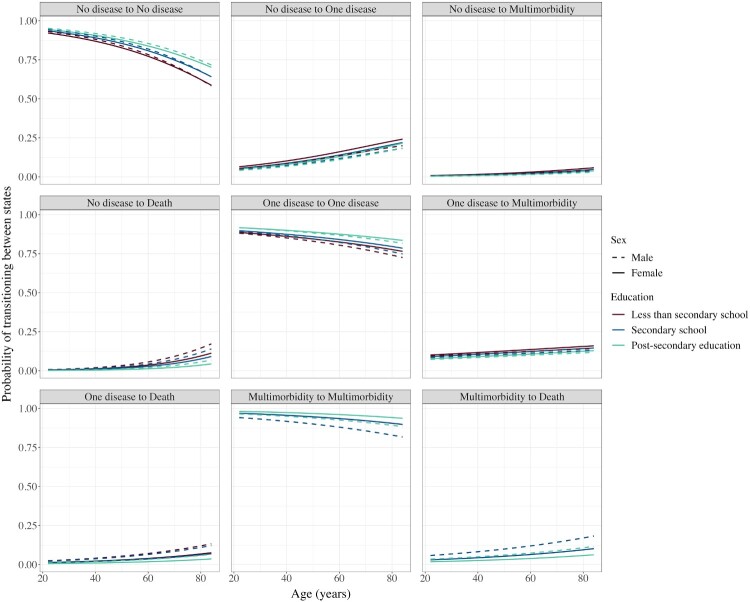
Probabilities of remaining in the same origin disease state or transitioning to a subsequent disease state or death over time, by sex and education *Note*: For the transitions from multimorbidity to multimorbidity and from multimorbidity to death, the lines for ‘Less than secondary school’ overlap almost exactly with those for ‘Secondary school’. *Source*: As for Figure A1.

Across races, the Asian/Indian and white groups are generally most likely to remain in the same disease state and least likely to transition to death (Appendix Figures A1 and A2). The opposite is true for the African and Coloured groups, particularly the males, who are most likely to transition to death from all states and least likely to remain in the same disease state. Coloured females show the highest probability of transitioning from no disease to one disease, and Asian/Indian people display the highest probability of transition from one disease to multimorbidity. In terms of educational differences, individuals with post-secondary education exhibit higher probabilities of remaining in the same disease state than those with less education, whereas less educated individuals are more likely to transition to death (Appendix Figure A3). Across both race and education, males display higher probabilities of death than females, while females are generally more likely to remain in the same disease state.

### Life expectancy at age 20

Based on our estimates for the period 2008–17, the average LE for females at age 20 is 44.3 years (95 per cent CI: 43.4–45.1), and the average LE for males is 37.6 years (95 per cent CI: 36.7–38.4) (Model 1, Table 2). This pattern of LE being higher for females than males is consistent across all races and education levels, although at different magnitudes. The white group and the post-secondary educated exhibit the highest life expectancies for both males and females (Models 2 and 3). WHO life table estimates for 2010 are higher for males (39.0 years) but lower for females (43.1 years) than our estimates (World Health Organization 2020).

**Table 2:** Weighted life expectancy at age 20, multimorbid life expectancy, and percentage of life expectancy spent with multimorbidity: South Africa, 2008–17

		Male			Female		
		*Weighted LE (95 per cent CI)*	*MMLE (95 per cent CI)*	*Percentage LE with MM*	*Weighted LE (95 per cent CI)*	*MMLE (95 per cent CI)*	*Percentage LE with MM*
*Model 1: Adjusted for age + residence, stratified by sex*		37.6 (36.7, 38.4)	9.8 (9.0, 10.6)	26	44.3 (43.4, 45.1)	17.3 (16.4, 18.1)	39
*Model 2: Model 1 + race*							
African		36.7 (35.8, 37.6)	9.0 (8.2, 9.7)	25	43.6 (42.7, 44.5)	16.5 (15.6, 17.4)	38
Asian/Indian		44.1 (38.5, 49.7)	13.4 (8.5, 18.4)	30	50.3 (45.5, 55.1)	21.5 (16.1, 26.9)	43
Coloured		38.5 (36.9, 40.1)	12.0 (10.5, 13.5)	31	45.9 (44.3, 47.5)	20.3 (18.6, 22.0)	44
White		46.5 (43.7, 49.3)	13.1 (10.5, 15.7)	28	52.1 (49.8, 54.5)	20.6 (17.7, 23.5)	40
*Model 3: Model 1 + education*							
Less than secondary school		35.3 (34.1, 36.4)	9.9 (9.0, 10.9)	28	42.6 (41.5, 43.6)	17.8 (16.7, 18.9)	42
Secondary school		37.6 (36.4, 38.9)	9.3 (8.2, 10.3)	25	44.3 (43.0, 45.6)	16.7 (15.4, 18.0)	38
Post-secondary education		46.4 (44.1, 48.7)	12.6 (10.6, 14.7)	27	51.9 (50.0, 53.8)	19.8 (17.5, 22.0)	38
*Model 4: Model 1 + interaction*							
African ×	Less than secondary school	35.5 (34.3, 36.7)	9.7 (8.7, 10.7)	27	42.8 (41.6, 43.9)	17.6 (16.5, 18.8)	41
	Secondary school or more	37.2 (35.9, 38.4)	8.2 (7.2, 9.2)	22	43.7 (42.4, 45.0)	15.3 (14.0, 16.6)	35
Coloured ×	Less than secondary school	35.0 (33.1, 36.9)	11.1 (9.4, 12.7)	32	42.9 (40.9, 44.8)	19.5 (17.5, 21.6)	45
	Secondary school or more	45.3 (42.1, 48.4)	16.0 (13.0, 18.9)	35	51.6 (49.1, 54.2)	24.0 (21.0, 26.9)	47
White ×	Less than secondary school	44.8 (33.9, 55.7)	19.2 (8.3, 30.2)	43	51.6 (43.0, 60.2)	28.2 (17.7, 38.8)	55
	Secondary school or more	46.1 (43.1, 49.0)	13.0 (10.2, 15.7)	28	51.7 (49.2, 54.2)	20.5 (17.4, 23.5)	40

*Notes:* Secondary school or more also includes individuals with post-secondary education. LE = life expectancy; MM = multimorbidity; MMLE = multimorbid life expectancy; CI = confidence interval.

*Source:* As for Table 1.

### Multimorbid life expectancy at age 20

**Figure 4:**
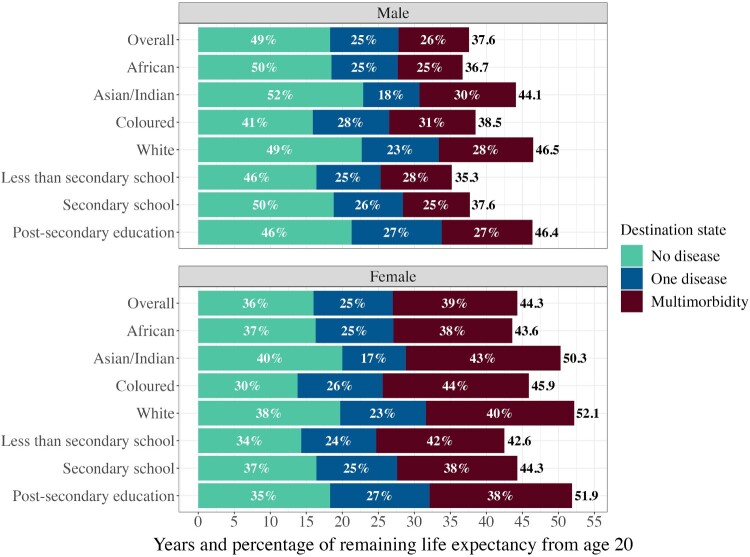
Weighted life expectancy from age 20 split by time spent in each state, overall and for each race and education group: males and females, South Africa, 2008–17 *Note*: Estimates are obtained from Models 1, 2, and 3, Table 2. Shaded bars show percentages of remaining life expectancy in each state; total weighted life expectancy in years is shown at the end of each bar. *Source*: As for Figure 2.

As expected, females experience higher MMLE and spend a greater fraction of their life with multimorbidity than males (17.3 years (39 per cent) vs 9.8 years (26 per cent)) (Table 2; Figure 4). This means they spend more time with at least two of the following diseases: Alzheimer’s disease, arthritis, asthma, cancer, diabetes, emphysema, epilepsy, heart problems, HIV, hypertension, kidney problems, psychological/psychiatric disorders, stroke, and tuberculosis. This pattern is observed regardless of race or education, with females consistently spending around 40 per cent of their remaining LE living with multimorbidity. For both males and females, MMLE is highest among the Asian/Indian group followed by the white, Coloured, then African groups (males 13.4, 13.1, 12.0, and 9.0 years, respectively; females 21.5, 20.6, 20.3, and 16.5 years, respectively). This finding is contrary to our hypothesis that the highest MMLE would be seen in the African group.

The pattern we observe across education is also contrary to our original expectation that higher levels of SES will be associated with lower MMLE. We observe the opposite pattern and find no education gradient. MMLE is highest among the post-secondary educated (males 12.6 years; females 19.8 years), but the group with less than secondary school education spends the greatest proportion of their LE with multimorbidity (males 28 per cent; females 42 per cent) (Model 3, Table 2). Those with less than secondary school education display the next highest MMLE among males (9.9 years) and females (17.8 years). The lowest MMLE belongs to those with secondary school education: 9.3 years for males and 16.7 years for females.

### Patterns of intersectionality and cumulative advantage/disadvantage

Looking at the intersection of race and sex, we observe similarly large sex differences across all races, with differences in MMLE ranging from 7.5 years in the African and white groups to 8.1 years in the Asian/Indian group and 8.3 years in the Coloured group (Model 2, Table 2). Similar magnitudes of differences are seen for sex and education. The sex gap in MMLE is slightly higher among the least educated (7.9 years) compared with those who have a secondary school (7.4 years) or post-secondary education (7.2 years) (Model 3, Table 2).

**Figure 5:**
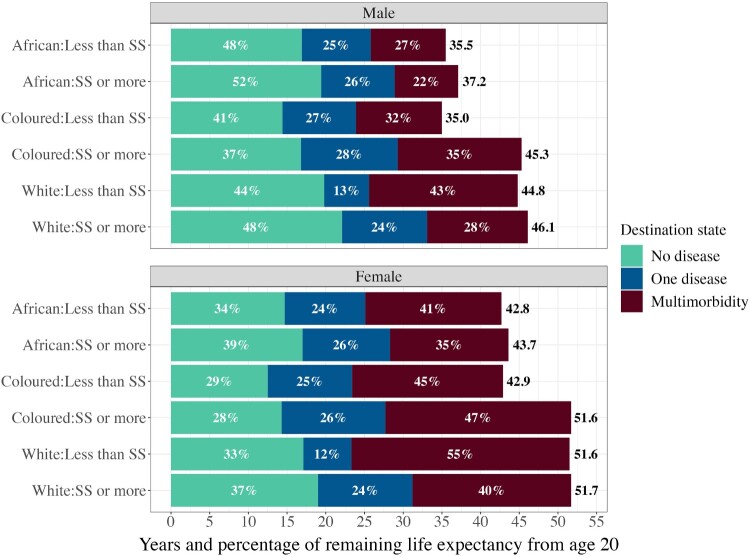
Weighted life expectancy from age 20 split by time spent in each state, overall and for each interacted race and dichotomized education group: males and females, South Africa, 2008–17 *Notes*: Estimates are obtained from Model 4, Table 2. Less than SS = less than secondary school; SS or more = secondary school or post-secondary education. Shaded bars show percentages of remaining life expectancy in each state; total weighted life expectancy in years is shown at the end of each bar. *Source*: As for Figure 2.

The interaction between race and dichotomized education shows that for males, the more highly educated African group experiences the lowest MMLE (8.2 years) and spends the smallest fraction of LE with multimorbidity (22 per cent), while the less educated Coloured group exhibits the lowest LE (35.0 years) (Figure 5; Model 4, Table 2). In contrast, less educated white males display the highest MMLE (19.2 years) and spend the greatest share of their LE with multimorbidity (43 per cent), while more highly educated white males show the longest LE (46.1 years) but spend only 28 per cent of their LE with multimorbidity. Life expectancy is 10.3 years longer for more highly educated Coloured males compared with their less educated counterparts. This difference is only 1.7 years for the African group and 1.3 years for the white group. While MMLE for more highly educated African and white males is 1.5 and 6.2 years lower, respectively, than for their less educated counterparts, for Coloured males MMLE among the more highly educated is 4.9 years *higher*. Thus, there is no clear evidence for cumulative advantage in males.

For females, we observe similar patterns. More highly educated African females exhibit both the lowest MMLE (15.3 years) and lowest fraction of LE with multimorbidity (35 per cent). Less educated white females display the highest MMLE (28.2 years, 55 per cent of LE). Coloured females seem to benefit the most from longer education by gaining the most years of life. Compared with the less educated group, more highly educated Coloured females gain 8.7 years of LE, while more highly educated African females gain 0.9 years and more highly educated white females gain only 0.1 years. MMLE is 4.5 years greater for more highly educated Coloured females than less educated Coloured females. In contrast, more highly educated African and white females experience *lower* MMLE than their less educated counterparts, with differences of 2.3 and 7.7 years, respectively. Therefore, we also find no evidence for cumulative advantage in females.

### Sensitivity analyses

Section IV (supplementary material) provides the origin disease state distribution and the state and LE estimates for all sensitivity analyses and also sample characteristics for the age 40+ analysis. These showed that excluding hypertension, tuberculosis, and both hypertension and tuberculosis from the definition of multimorbidity (parts 1–3, respectively) did not result in major changes to LE compared with the main analysis (Section III, supplementary material); males and females generally gained slightly more LE when these diseases were excluded. There were noticeable decreases in MMLE, particularly when hypertension was excluded, either individually or with tuberculosis, as expected from the high prevalence of hypertension in the sample. These patterns were observed across all race and education groups. When we conducted analyses for participants aged 40+ (part 4), we found that the same general trends were also observed. However, there was a shift in the distribution of disease, with more people beginning from an origin state of multimorbidity and spending the majority of their LE with multimorbidity.

## Discussion

To our knowledge, this has been the first paper to use an incidence-based multistate modelling approach to estimate LE and MMLE in South Africa. We also introduced the term ‘multimorbid life expectancy’ to promote the use of consistent terminology in this field. Using an intersectionality and cumulative advantage/disadvantage framework, we examined the relationship between race, sex, and education—as independent and intersecting factors—and MMLE in South Africa.

We found that females experienced both higher LE and higher MMLE than males in South Africa. This pattern was consistent across all race and education groups, and the difference in MMLE between females and males was steady at around eight years in all groups. We also observed that Asian/Indian and white groups and the post-secondary educated displayed the highest LE and MMLE. Observed patterns across absolute and relative time spent with multimorbidity generally remained consistent between groups. However, in some cases, although the proportion of time with multimorbidity may have been similar, the absolute years differed due to the wide variation in LE. Lastly, we did not find evidence for cumulative advantage, but we did identify an interesting pattern in our race and education interaction. The educational gain in LE was highest among the Coloured group (compared with the African and white groups), but the Coloured group was the only one that saw an accompanying increase in MMLE. It is unclear whether this increase in MMLE alongside an increase in LE should be viewed positively or negatively.

Multimorbidity is a complex outcome, especially so when considered in conjunction with LE. It is clear that higher MMLE is worse than lower MMLE, that is, spending more years suffering with multiple diseases is detrimental for individuals and societies. By contrast, higher LE is better than lower LE. Considering MMLE and LE simultaneously, however, leads to ambiguity in the interpretation: it is not always clear which combinations of LE and MMLE might be considered better or worse outcomes. It is easy to identify that the highly discordant pairs (low MMLE & high LE and high MMLE & low LE) correspond to the best and worst outcomes, respectively. However, the more concordant pairs (low MMLE & low LE and high MMLE & high LE) are where uncertainty lies. This is important, because most of our results were concordant pairs; groups with higher LE tended to exhibit higher MMLE and groups with lower LE tended to exhibit lower MMLE.

This is of particular relevance when thinking about MMLE and LE within a cumulative advantage/disadvantage framework, because it is unclear which scenario will be more advantageous or disadvantageous. People with higher MMLE and LE likely have less severe conditions or more resources to access and manage their care. Thus, they can live longer with more disease and could be considered advantaged. This is unsurprising, as there is a well-established socio-economic gradient in healthy LE (Crimmins and Hagedorn 2010; Pongiglione et al. 2015; Islam et al. 2018). In contrast, those with both low MMLE and low LE may die earlier due to having diseases with higher fatality rates or potentially lacking the resources to manage their morbidities even though they experience less disease overall, and thus could be considered disadvantaged. However, some might argue that living longer, regardless of disease status, represents a more advantaged status. However, our perspective might also depend on whether we consider questions on disease severity, disability, and quality of life. Moreover, this discussion leads us to question the widely held notion that more disease is necessarily a worse outcome. As multimorbidity rates continue to increase, this will become an even more important question, requiring further research.

Another approach to understanding the meaning of MMLE better would be to disaggregate multimorbidity into disease types, number of diseases, and whether conditions are controlled or progressively worsening. These characteristics would allow for a better understanding of the severity of multimorbidity and its impact on LE and quality of life. More severe and less manageable multimorbidity is likely a main contributor to lower MMLE and LE, and the reverse could also be true. If it is not feasible to disentangle multimorbidity into such a detailed form, another option would be to consider disability within the context of multimorbidity. It could act as a proxy for the severity of multimorbidity and would allow a more nuanced understanding of how much time someone could spend with mild vs severe disease. This is also relevant because multimorbidity is beginning to develop at younger ages, likely due to a combination of changing health behaviours (e.g. alcohol consumption, obesity) and improved disease screening and treatment (Bishop et al. 2022). If younger people manage their multimorbidity well, then they should be able to live longer without much detriment.

The increasingly earlier incidence of multimorbidity provides one reason for future researchers to consider taking a life-course approach. This is especially pertinent in South Africa, due to its apartheid history playing a significant role in people’s upbringing and access to resources and opportunities. Apartheid has likely had different effects on individuals, dependent on their social position but also on how much of the apartheid regime they lived through. The effect of apartheid is likely very different for someone who was born prior to and lived most their life under apartheid compared with someone born towards the end. One major difference between these groups would be their access to education due to systemic racial segregation during apartheid (Meek and Meek 2008). The significance of certain educational levels likely differs due to the range of availability and quality of education across racial groups and birth cohorts. This is supported by our findings, which showed that African and Coloured people tended to be less educated and consistently displayed lower MMLE and LE compared with Asian/Indian and white people. However, we observed that Coloured males and females gained more LE from a higher level of education than other racial groups, and they were also the only group where the more highly educated showed greater MMLE than the less educated. It is unclear why education plays such a substantial and unique role in LE and MMLE in the Coloured group, but it may be due to differences in health behaviours or educational opportunities over time. There are also likely structural differences in opportunities and systematic biases that are deeply embedded in South African society. It would be interesting in future research to conduct life-course and cohort analyses to see how the relationships between race, education, and multimorbidity might differ over time.

This study has several limitations. First, our LE estimate for males was lower than that provided by the World Health Organization, whereas our LE estimate for females was higher. This could be due to the males in our sample being unhealthier than the general population and the females in our sample being healthier. There might also be confounding factors which were not accounted for in the analysis. The second limitation is that the disease data were self-reported, which makes them prone to recall bias, and we did not have sufficient information to determine whether an individual continued to suffer from that disease. It is possible for people to be cured of certain diseases, but the chronic conditions we included in our study can also have long-term effects on health, even if the individual is technically cured. For example, the likelihood of being microbiologically cured from tuberculosis is high, but many individuals still experience persistent symptoms (i.e. post-tuberculosis lung disease) and social, psychological, and economic consequences (Allwood et al. 2021). Third, the questionnaires asked about only a certain number of diseases, thus limiting what we could include within our definition of multimorbidity. Thus, although we classified people as having ‘no disease’, they might have had one or more diseases that were not included in our definition. There is also potential for underdiagnosis of disease, so participants may have had one or more diseases but just not have been diagnosed. This is also likely correlated with SES, as groups with higher SES are more likely to seek and access care compared with lower SES groups. Both these instances would result in an underestimate of multimorbidity that might vary by social group. Factors which we were not able to account for in this paper (e.g. healthcare accessibility) might help to explain these intersectional differences. Lastly, there were quite small sample sizes in some strata, particularly for the Asian/Indian group and when looking at the interaction between race and education. This required us either to combine strata or to exclude groups altogether. However, we were still left with some small groups in our analysis, resulting in very wide confidence intervals, specifically in the less educated white group. These smaller samples in the Asian/Indian and white groups were also related to the attrition patterns seen throughout the data. This highlights the need for larger and more representative studies to ensure sufficient participants even in the smaller strata.

In this study, we found that race, SES, and sex showed independent and intersectional associations with MMLE in South Africa. We did not find any evidence for cumulative advantage or disadvantage but identified substantial inequalities in both LE and MMLE across race and education, with African males and females facing the most disadvantages, even at higher levels of education. This demonstrates that the racial and socio-economic hierarchies in place during apartheid have had lasting impacts on life-course health disparities, highlighting entrenched structural inequalities. As South Africa is beginning to implement a national health insurance system (Murphy and Moosa 2021; Pauw 2022), it is timely for actions to be taken to address these inequalities. Existing systems tend to be fragmented and were built to treat acute diseases, but people with NCDs and multimorbidity require different types of care (Basto-Abreu et al. 2022). There is a need to redesign health systems to care properly for patients with multimorbidity and to overcome the structural inequalities that discourage and prevent certain groups of people from accessing health services. Efforts should also be made to develop a more equal education system and provide necessary educational resources to those in need. These recommendations revolve around the idea of proportionate universalism, in which actions should be implemented universally but to an extent proportional to the level of disadvantage (Marmot and Bell 2012).

## Supplementary Material

Supplemental Material
