# Supplementary material for: Multimorbid life expectancy across race, socio-economic status, and sex in South Africa
Source: Popul Stud (Camb). 2024 May 16;79(1):1–26. doi: 10.1080/00324728.2024.2331447 (PMC11956785; doi:10.1080/00324728.2024.2331447)
Supplement: Supplemental Material [file RPST_A_2331447_SM9837.pdf]

Supplementary Material

Multimorbid life expectancy across race, socio-economic status, and sex in South Africa

Anastasia Lam[1,2], Katherine Keenan[1], Mikko Myrskylä[2,3], Hill Kulu[1]

[1]University of St Andrews, [2]Max Planck Institute for Demographic Research,

[3]University of Helsinki

Table of Contents

Section I: Excluded vs included sample comparison 2

Section II: Included multimorbid conditions...3

Section III: Complete expectancy estimates with 95% confidence intervals..........................4

Section IV: Sensitivity analyses..10

Section I: Excluded vs included sample comparison

	Excluded (N=10,207)	Included (N=18,030)	p-value
**Sex**			< 0.001
Female	5,583 (54.7%)	10,806 (59.9%)	
Male	4,624 (45.3%)	7,224 (40.1%)	
N missing	0	0	
**Age (years)**			< 0.001
Mean (SD)	44.5 (16.1)	42.0 (16.2)	
Range	20.0–106	20.0–106	
N missing	635 (6.2%)	0	
**Education**			< 0.001
Less than secondary school	3,041 (29.8%)	8,330 (46.2%)	
Secondary school	4,866 (47.7%)	7,500 (41.6%)	
Post-secondary education	2,098 (20.6%)	2,200 (12.2%)	
N missing	202 (2.0%)	0	
**Race**			< 0.001
African	6,989 (68.5%)	14,410 (79.9%)	
Asian/Indian	379 (3.7%)	225 (1.2%)	
Coloured	1,285 (12.6%)	2,666 (14.8%)	
White	1,545 (15.1%)	729 (4.0%)	
N missing	9 (0.1%)	0	
**Residence**			< 0.001
Rural	3,752 (36.8%)	8,988 (49.9%)	
Urban	6,348 (62.2%)	9,042 (50.1%)	
N missing	107 (1.0%)	0	
**Origin disease state**			< 0.001
No disease	6,685 (65.5%)	12,571 (69.7%)	
One disease	1,835 (18.0%)	4,026 (22.3%)	
Multimorbidity	620 (6.1%)	1,433 (7.9%)	
N missing	1,067 (10.5%)	0	

Section II: Included multimorbid conditions

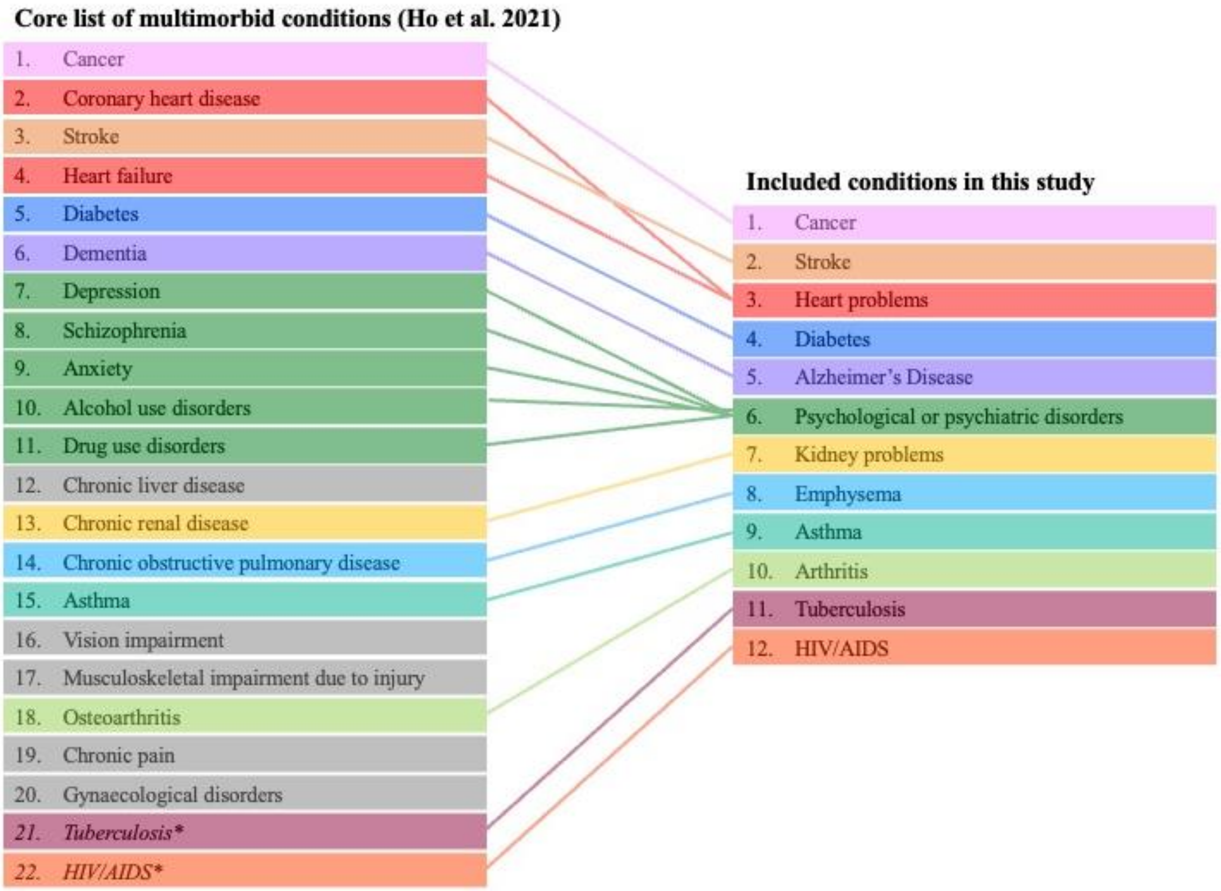

Figure S1. Comparison of multimorbid conditions included in this study with the core list from Ho et al. (2021)

Note: *Tuberculosis and HIV/AIDS are not included in Ho et al.'s list of 20 core conditions, but it was noted in the list that they could be included as core conditions for certain countries.

Section III: Complete expectancy estimates with 95% confidence intervals

Model 1: Stratified by sex

Male

	Origin state											
	No disease			One disease			Multimorbidity			Weighted average		
Destination state	Est.	LCI	UCI	Est.	LCI	UCI	Est.	LCI	UCI	Est.	LCI	UCI
No disease	19.8	19.2	20.3	0.0	0.0	0.0	0.0	0.0	0.0	18.3	17.7	18.8
One disease	9.2	8.7	9.7	14.8	14.0	15.6	0.0	0.0	0.0	9.5	9.0	10.0
Multimorbidity	9.3	8.6	10.1	14.8	13.5	16.0	23.4	21.6	25.2	9.8	9.0	10.6
Total	38.3	37.4	39.1	29.6	28.3	30.8	23.4	21.6	25.2	37.6	36.7	38.4

Female

	Origin state											
	No disease			One disease			Multimorbidity			Weighted average		
Destination state	Est.	LCI	UCI	Est.	LCI	UCI	Est.	LCI	UCI	Est.	LCI	UCI
No disease	18.3	17.8	18.8	0.0	0.0	0.0	0.0	0.0	0.0	16.0	15.6	16.5
One disease	10.6	10.2	11.0	15.4	14.7	16.2	0.0	0.0	0.0	11.0	10.5	11.4
Multimorbidity	16.2	15.4	16.9	23.7	22.4	25.0	34.5	32.7	36.2	17.3	16.4	18.1
Total	45.1	44.3	45.9	39.2	38.0	40.4	34.5	32.7	36.2	44.3	43.4	45.1

Model 2: Stratified by sex and race

Male, African

	Origin state											
	No disease			One disease			Multimorbidity			Weighted average		
Destination state	Est.	LCI	UCI	Est.	LCI	UCI	Est.	LCI	UCI	Est.	LCI	UCI
No disease	20.1	19.5	20.7	0.0	0.0	0.0	0.0	0.0	0.0	18.5	18.0	19.1
One disease	8.9	8.3	9.4	14.6	13.7	15.4	0.0	0.0	0.0	9.2	8.7	9.7
Multimorbidity	8.5	7.8	9.3	13.7	12.4	15.0	22	20.2	23.8	9.0	8.2	9.7
Total	37.4	36.5	38.3	28.3	27.0	29.6	22	20.2	23.8	36.7	35.8	37.6

Male, Asian/Indian

	Origin state											
	No disease			One disease			Multimorbidity			Weighted average		
Destination state	Est.	LCI	UCI	Est.	LCI	UCI	Est.	LCI	UCI	Est.	LCI	UCI
No disease	24.7	20.6	28.9	0.0	0.0	0.0	0.0	0.0	0.0	22.9	19.1	26.7
One disease	7.5	5.0	10.0	12.9	9.4	16.4	0.0	0.0	0.0	7.8	5.3	10.4
Multimorbidity	12.6	7.9	17.4	22.2	14.6	29.7	31.3	23	39.6	13.4	8.5	18.4
Total	44.9	39.4	50.4	35.1	27.8	42.3	31.3	23	39.6	44.1	38.5	49.7

Male, Coloured

	Origin state											
	No disease			One disease			Multimorbidity			Weighted average		
Destination state	Est.	LCI	UCI	Est.	LCI	UCI	Est.	LCI	UCI	Est.	LCI	UCI
No disease	17.2	16.3	18.1	0.0	0.0	0.0	0.0	0.0	0.0	15.9	15.1	16.8
One disease	10.3	9.4	11.2	15.4	14.2	16.6	0.0	0.0	0.0	10.6	9.7	11.5
Multimorbidity	11.5	10.1	12.9	16.9	14.9	18.9	26.2	23.7	28.8	12.0	10.5	13.5
Total	39.0	37.4	40.7	32.3	30.3	34.3	26.2	23.7	28.8	38.5	36.9	40.1

Male, White

	Origin state											
	No disease			One disease			Multimorbidity			Weighted average		
Destination state	Est.	LCI	UCI	Est.	LCI	UCI	Est.	LCI	UCI	Est.	LCI	UCI
No disease	24.5	22.3	26.8	0.0	0.0	0.0	0.0	0.0	0.0	22.7	20.6	24.8
One disease	10.4	8.8	12.0	16.8	14.5	19.1	0.0	0.0	0.0	10.7	9.1	12.4
Multimorbidity	12.4	9.9	14.9	21.3	17.2	25.3	32.2	27.5	37	13.1	10.5	15.7
Total	47.3	44.5	50.0	38.1	34.3	41.9	32.2	27.5	37	46.5	43.7	49.3

Female, African

	Origin state											
	No disease			One disease			Multimorbidity			Weighted average		
Destination state	Est.	LCI	UCI	Est.	LCI	UCI	Est.	LCI	UCI	Est.	LCI	UCI
No disease	18.6	18.1	19.1	0.0	0.0	0.0	0.0	0.0	0.0	16.3	15.8	16.7
One disease	10.4	10.0	10.9	15.3	14.5	16.1	0.0	0.0	0.0	10.8	10.3	11.3
Multimorbidity	15.4	14.6	16.2	22.8	21.5	24.2	33.3	31.5	35.1	16.5	15.6	17.4
Total	44.4	43.6	45.3	38.1	36.9	39.4	33.3	31.5	35.1	43.6	42.7	44.5

Female, Asian/Indian

	Origin state											
	No disease			One disease			Multimorbidity			Weighted average		
Destination state	Est.	LCI	UCI	Est.	LCI	UCI	Est.	LCI	UCI	Est.	LCI	UCI
No disease	22.9	18.9	26.9	0.0	0.0	0.0	0.0	0.0	0.0	20.0	16.5	23.5
One disease	8.4	5.7	11.1	13.0	9.4	16.6	0.0	0.0	0.0	8.8	6.1	11.5
Multimorbidity	19.8	14.6	25.0	32.1	24.8	39.5	42.7	35.3	50.2	21.5	16.1	26.9
Total	51.1	46.5	55.7	45.2	38.8	51.5	42.7	35.3	50.2	50.3	45.5	55.1

Female, Coloured

	Origin state											
	No disease			One disease			Multimorbidity			Weighted average		
Destination state	Est.	LCI	UCI	Est.	LCI	UCI	Est.	LCI	UCI	Est.	LCI	UCI
No disease	15.7	14.9	16.6	0.0	0.0	0.0	0.0	0.0	0.0	13.8	13.0	14.5
One disease	11.6	10.6	12.5	15.8	14.6	17.1	0.0	0.0	0.0	11.8	10.9	12.8
Multimorbidity	19.2	17.5	20.8	26.4	24.2	28.5	37.8	35.3	40.4	20.3	18.6	22.0
Total	46.5	45.0	48.0	42.2	40.3	44.1	37.8	35.3	40.4	45.9	44.3	47.5

Female, White

	Origin state											
	No disease			One disease			Multimorbidity			Weighted average		
Destination state	Est.	LCI	UCI	Est.	LCI	UCI	Est.	LCI	UCI	Est.	LCI	UCI
No disease	22.5	20.3	24.7	0.0	0.0	0.0	0.0	0.0	0.0	19.7	17.8	21.5
One disease	11.5	9.7	13.2	17.0	14.6	19.4	0.0	0.0	0.0	11.9	10.1	13.7
Multimorbidity	18.9	16.2	21.7	30.4	26.4	34.4	43.5	39.3	47.7	20.6	17.7	23.5
Total	52.9	50.6	55.1	47.4	44.2	50.6	43.5	39.3	47.7	52.1	49.8	54.5

Model 3: Stratified by sex and education

Male, Less than secondary school

	Origin state											
	No disease			One disease			Multimorbidity			Weighted average		
Destination state	Est.	LCI	UCI	Est.	LCI	UCI	Est.	LCI	UCI	Est.	LCI	UCI
No disease	17.8	17.0	18.5	0.0	0.0	0.0	0.0	0.0	0.0	16.4	15.7	17.1
One disease	8.7	8.1	9.3	13.5	12.6	14.5	0.0	0.0	0.0	8.9	8.3	9.6
Multimorbidity	9.5	8.6	10.4	14.5	13.0	16.0	22.6	20.6	24.6	9.9	9.0	10.9
Total	35.9	34.8	37.0	28.0	26.5	29.5	22.6	20.6	24.6	35.3	34.1	36.4

Male, Secondary school

	Origin state											
	No disease			One disease			Multimorbidity			Weighted average		
Destination state	Est.	LCI	UCI	Est.	LCI	UCI	Est.	LCI	UCI	Est.	LCI	UCI
No disease	20.3	19.6	21.0	0.0	0.0	0.0	0.0	0.0	0.0	18.8	18.1	19.4
One disease	9.3	8.6	9.9	14.8	13.8	15.8	0.0	0.0	0.0	9.6	8.9	10.3
Multimorbidity	8.8	7.8	9.8	14.2	12.6	15.8	22.7	20.6	24.7	9.3	8.2	10.3
Total	38.4	37.1	39.6	29.0	27.4	30.6	22.7	20.6	24.7	37.6	36.4	38.9

Male, Post-secondary education

	Origin state											
	No disease			One disease			Multimorbidity			Weighted average		
Destination state	Est.	LCI	UCI	Est.	LCI	UCI	Est.	LCI	UCI	Est.	LCI	UCI
No disease	23.1	21.7	24.4	0.0	0.0	0.0	0.0	0.0	0.0	21.3	20.1	22.5
One disease	12.1	10.8	13.4	18.6	16.7	20.5	0.0	0.0	0.0	12.5	11.1	13.8
Multimorbidity	12.0	10.0	13.9	19.9	16.7	23.0	31.3	27.4	35.3	12.6	10.6	14.7
Total	47.1	44.9	49.4	38.4	35.4	41.5	31.3	27.4	35.3	46.4	44.1	48.7

Female, Less than secondary school

	Origin state											
	No disease			One disease			Multimorbidity			Weighted average		
Destination state	Est.	LCI	UCI	Est.	LCI	UCI	Est.	LCI	UCI	Est.	LCI	UCI
No disease	16.4	15.7	17.1	0.0	0.0	0.0	0.0	0.0	0.0	14.3	13.7	15.0
One disease	10.1	9.6	10.7	14.3	13.3	15.2	0.0	0.0	0.0	10.4	9.8	11.0
Multimorbidity	16.8	15.8	17.8	23.7	22.2	25.3	33.8	31.8	35.8	17.8	16.7	18.9
Total	43.3	42.3	44.3	38.0	36.6	39.4	33.8	31.8	35.8	42.6	41.5	43.6

Female, Secondary school

	Origin state											
	No disease			One disease			Multimorbidity			Weighted average		
Destination state	Est.	LCI	UCI	Est.	LCI	UCI	Est.	LCI	UCI	Est.	LCI	UCI
No disease	18.8	18.2	19.4	0.0	0.0	0.0	0.0	0.0	0	16.4	15.9	17.0
One disease	10.9	10.2	11.5	15.6	14.7	16.6	0.0	0.0	0	11.2	10.5	11.9
Multimorbidity	15.5	14.3	16.8	23.2	21.4	25.0	33.8	31.6	36	16.7	15.4	18.0
Total	45.2	44.0	46.4	38.8	37.1	40.4	33.8	31.6	36	44.3	43.0	45.6

Female, Post-secondary education

	Origin state											
	No disease			One disease			Multimorbidity			Weighted average		
Destination state	Est.	LCI	UCI	Est.	LCI	UCI	Est.	LCI	UCI	Est.	LCI	UCI
No disease	21.0	19.8	22.2	0.0	0.0	0.0	0.0	0.0	0.0	18.3	17.3	19.4
One disease	13.4	12.0	14.8	18.9	17.0	20.9	0.0	0.0	0.0	13.8	12.4	15.2
Multimorbidity	18.2	16.1	20.4	28.4	25.3	31.6	42.5	39	46.1	19.8	17.5	22.0
Total	52.7	50.8	54.5	47.4	44.8	50.0	42.5	39	46.1	51.9	50.0	53.9

Model 4: Stratified by sex and race*binary education interaction

Male, African, Less than secondary school

	Origin state											
	No disease			One disease			Multimorbidity			Weighted average		
Destination state	Est.	LCI	UCI	Est.	LCI	UCI	Est.	LCI	UCI	Est.	LCI	UCI
No disease	18.3	17.5	19.1	0.0	0.0	0.0	0.0	0.0	0.0	16.9	16.1	17.7
One disease	8.6	8.0	9.2	13.6	12.6	14.6	0.0	0.0	0.0	8.9	8.3	9.5
Multimorbidity	9.2	8.3	10.2	14.4	12.9	16.0	22.6	20.5	24.7	9.7	8.7	10.7
Total	36.1	35.0	37.3	28.0	26.4	29.6	22.6	20.5	24.7	35.5	34.3	36.7

Male, African, Secondary school or more

	Origin state											
	No disease			One disease			Multimorbidity			Weighted average		
Destination state	Est.	LCI	UCI	Est.	LCI	UCI	Est.	LCI	UCI	Est.	LCI	UCI
No disease	21.0	20.3	21.8	0.0	0.0	0.0	0.0	0.0	0.0	19.4	18.8	20.1
One disease	9.2	8.5	9.9	15.1	14.1	16.1	0.0	0.0	0.0	9.5	8.9	10.2
Multimorbidity	7.7	6.8	8.7	12.9	11.4	14.4	21.2	19.2	23.2	8.2	7.2	9.2
Total	38.0	36.7	39.2	28.0	26.4	29.5	21.2	19.2	23.2	37.2	35.9	38.4

Male, Coloured, Less than secondary school

	Origin state											
	No disease			One disease			Multimorbidity			Weighted average		
Destination state	Est.	LCI	UCI	Est.	LCI	UCI	Est.	LCI	UCI	Est.	LCI	UCI
No disease	15.6	14.4	16.8	0.0	0.0	0.0	0	0.0	0.0	14.4	13.3	15.5
One disease	9.3	8.3	10.3	13.8	12.4	15.1	0	0.0	0.0	9.5	8.5	10.6
Multimorbidity	10.6	9.0	12.2	15.5	13.2	17.7	24	21.2	26.7	11.1	9.4	12.7
Total	35.5	33.6	37.4	29.2	27.0	31.5	24	21.2	26.7	35.0	33.1	36.9

Male, Coloured, Secondary school or more

	Origin state											
	No disease			One disease			Multimorbidity			Weighted average		
Destination state	Est.	LCI	UCI	Est.	LCI	UCI	Est.	LCI	UCI	Est.	LCI	UCI
No disease	18.2	16.9	19.5	0.0	0.0	0.0	0.0	0.0	0.0	16.8	15.6	18.0
One disease	12.2	10.6	13.8	17.2	15.1	19.2	0.0	0.0	0.0	12.5	10.9	14.1
Multimorbidity	15.3	12.5	18.2	22.6	18.6	26.7	34.1	29.3	38.9	16.0	13.0	18.9
Total	45.8	42.7	48.8	39.8	35.9	43.6	34.1	29.3	38.9	45.3	42.1	48.4

Male, White, Less than secondary school

	Origin state											
	No disease			One disease			Multimorbidity			Weighted average		
Destination state	Est.	LCI	UCI	Est.	LCI	UCI	Est.	LCI	UCI	Est.	LCI	UCI
No disease	21.5	12.3	30.6	0.0	0.0	0.0	0.0	0	0.0	19.8	11.4	28.3
One disease	5.6	2.2	9.0	9.0	4.7	13.4	0.0	0	0.0	5.8	2.4	9.2
Multimorbidity	18.3	7.5	29.1	29.7	15.3	44.1	37.6	23	52.3	19.2	8.3	30.2
Total	45.4	34.6	56.1	38.7	25.1	52.4	37.6	23	52.3	44.8	33.9	55.7

Male, White, Secondary school or more

	Origin state											
	No disease			One disease			Multimorbidity			Weighted average		
Destination state	Est.	LCI	UCI	Est.	LCI	UCI	Est.	LCI	UCI	Est.	LCI	UCI
No disease	23.9	21.6	26.3	0.0	0.0	0.0	0.0	0.0	0.0	22.1	19.9	24.3
One disease	10.7	9.0	12.4	16.8	14.3	19.2	0.0	0.0	0.0	11.0	9.3	12.8
Multimorbidity	12.2	9.6	14.9	20.9	16.7	25.2	31.8	26.8	36.8	13.0	10.2	15.7
Total	46.8	43.9	49.7	37.7	33.8	41.7	31.8	26.8	36.8	46.1	43.1	49.0

Female, African, Less than secondary school

	Origin state											
	No disease			One disease			Multimorbidity			Weighted average		
Destination state	Est.	LCI	UCI	Est.	LCI	UCI	Est.	LCI	UCI	Est.	LCI	UCI
No disease	16.9	16.1	17.6	0.0	0.0	0.0	0.0	0.0	0	14.7	14.1	15.4
One disease	10.1	9.5	10.7	14.3	13.3	15.2	0.0	0.0	0	10.4	9.8	11.0
Multimorbidity	16.5	15.5	17.6	23.8	22.1	25.4	33.9	31.8	36	17.6	16.5	18.8
Total	43.5	42.4	44.6	38.0	36.6	39.5	33.9	31.8	36	42.8	41.6	43.9

Female, African, Secondary school or more

	Origin state											
	No disease			One disease			Multimorbidity			Weighted average		
Destination state	Est.	LCI	UCI	Est.	LCI	UCI	Est.	LCI	UCI	Est.	LCI	UCI
No disease	19.5	18.8	20.1	0.0	0.0	0.0	0.0	0.0	0.0	17.0	16.5	17.6
One disease	11.0	10.3	11.7	16.0	15.0	17.0	0.0	0.0	0.0	11.3	10.6	12.0
Multimorbidity	14.2	12.9	15.4	21.7	19.9	23.5	32.3	30.1	34.5	15.3	14.0	16.6
Total	44.6	43.4	45.9	37.7	36.0	39.3	32.3	30.1	34.5	43.7	42.4	45.0

Female, Coloured, Less than secondary school

	Origin state											
	No disease			One disease			Multimorbidity			Weighted average		
Destination state	Est.	LCI	UCI	Est.	LCI	UCI	Est.	LCI	UCI	Est.	LCI	UCI
No disease	14.3	13.1	15.4	0.0	0.0	0.0	0.0	0.0	0.0	12.5	11.5	13.5
One disease	10.6	9.5	11.8	14.4	13.0	15.8	0.0	0.0	0.0	10.9	9.8	12.0
Multimorbidity	18.5	16.5	20.5	25.0	22.4	27.6	35.4	32.5	38.3	19.5	17.5	21.6
Total	43.4	41.6	45.3	39.4	37.1	41.6	35.4	32.5	38.3	42.9	40.9	44.8

Female, Coloured, Secondary school or more

	Origin state											
	No disease			One disease			Multimorbidity			Weighted average		
Destination state	Est.	LCI	UCI	Est.	LCI	UCI	Est.	LCI	UCI	Est.	LCI	UCI
No disease	16.3	15.2	17.5	0.0	0.0	0.0	0.0	0.0	0.0	14.3	13.2	15.3
One disease	13.2	11.5	14.8	17.3	15.2	19.3	0.0	0.0	0.0	13.4	11.7	15.1
Multimorbidity	22.6	19.8	25.5	31.5	27.7	35.2	45.1	41.1	49.1	24.0	21.0	26.9
Total	52.1	49.6	54.6	48.7	45.6	51.9	45.1	41.1	49.1	51.6	49.1	54.2

Female, White, Less than secondary school

	Origin state											
	No disease			One disease			Multimorbidity			Weighted average		
Destination state	Est.	LCI	UCI	Est.	LCI	UCI	Est.	LCI	UCI	Est.	LCI	UCI
No disease	19.6	10.8	28.3	0.0	0.0	0.0	0.0	0.0	0.0	17.1	9.5	24.8
One disease	6.0	2.5	9.6	8.9	4.6	13.3	0.0	0.0	0.0	6.2	2.7	9.8
Multimorbidity	26.5	15.8	37.1	39.6	27.6	51.6	47.9	36.6	59.3	28.2	17.7	38.8
Total	52.1	43.7	60.4	48.5	37.9	59.2	47.9	36.6	59.3	51.6	43.0	60.2

Female, White, Secondary school or more

	Origin state											
	No disease			One disease			Multimorbidity			Weighted average		
Destination state	Est.	LCI	UCI	Est.	LCI	UCI	Est.	LCI	UCI	Est.	LCI	UCI
No disease	21.8	19.5	24.0	0	0.0	0.0	0.0	0.0	0.0	19.0	17.1	21.0
One disease	11.9	10.0	13.7	17	14.4	19.6	0.0	0.0	0.0	12.2	10.3	14.1
Multimorbidity	18.8	15.9	21.8	30	25.8	34.3	43.1	38.7	47.6	20.5	17.4	23.5
Total	52.5	50.1	54.9	47	43.6	50.4	43.1	38.7	47.6	51.7	49.2	54.2

Section IV: Sensitivity analyses

1 Exclude hypertension from multimorbidity definition

1.1 Origin disease state distribution

Origin disease state	Male (n=7224)	Female (n=10,806)	Overall (N=18,030)
No disease	6,213 (86.0%)	8,823 (81.6%)	15,036 (83.4%)
One disease	877 (12.1%)	1,657 (15.3%)	2,534 (14.1%)
Multimorbidity	134 (1.9%)	326 (3.0%)	460 (2.6%)

1.2 Expectancy estimates

Model 1: Stratified by sex

Male

	Origin state											
	No disease			One disease			Multimorbidity			Weighted average		
Destination state	Est.	LCI	UCI	Est.	LCI	UCI	Est.	LCI	UCI	Est.	LCI	UCI
No disease	25.8	25.1	26.6	0.0	0.0	0.0	0.0	0.0	0.0	24.5	23.8	25.2
One disease	7.9	7.4	8.4	16.4	15.3	17.5	0.0	0.0	0.0	8.3	7.8	8.8
Multimorbidity	4.6	4.1	5.2	10.0	8.7	11.3	20.3	18.1	22.4	5.0	4.3	5.6
Total	38.4	37.5	39.2	26.4	25.0	27.7	20.3	18.1	22.4	37.7	36.9	38.6

Female

	Origin state											
	No disease			One disease			Multimorbidity			Weighted average		
Destination state	Est.	LCI	UCI	Est.	LCI	UCI	Est.	LCI	UCI	Est.	LCI	UCI
No disease	25.7	25.0	26.3	0.0	0.0	0.0	0.0	0.0	0.0	23.5	22.9	24.1
One disease	9.7	9.3	10.2	17.4	16.4	18.5	0.0	0.0	0.0	10.3	9.7	10.8
Multimorbidity	9.6	8.8	10.3	18.7	17.1	20.4	31.8	29.5	34.2	10.5	9.6	11.3
Total	45.0	44.2	45.8	36.2	34.7	37.7	31.8	29.5	34.2	44.2	43.3	45.1

Model 2: Stratified by sex and race

Male, African

	Origin state											
	No disease			One disease			Multimorbidity			Weighted average		
Destination state	Est.	LCI	UCI	Est.	LCI	UCI	Est.	LCI	UCI	Est.	LCI	UCI
No disease	25.8	25.0	26.5	0.0	0.0	0.0	0.0	0.0	0.0	24.4	23.7	25.2
One disease	7.6	7.0	8.1	16.0	14.9	17.0	0.0	0.0	0.0	7.9	7.4	8.5
Multimorbidity	4.3	3.7	4.8	9.3	8.0	10.6	19.3	17.1	21.4	4.6	4.0	5.2
Total	37.6	36.7	38.5	25.3	23.9	26.6	19.3	17.1	21.4	36.9	36.0	37.8

Male, Asian/Indian

	Origin state											
	No disease			One disease			Multimorbidity			Weighted average		
Destination state	Est.	LCI	UCI	Est.	LCI	UCI	Est.	LCI	UCI	Est.	LCI	UCI
No disease	27.5	22.8	32.2	0.0	0.0	0.0	0.0	0.0	0.0	26.1	21.6	30.5
One disease	9.5	6.2	12.8	18.3	13.3	23.3	0.0	0.0	0.0	9.9	6.6	13.2
Multimorbidity	7.1	3.2	11.0	14.9	7.5	22.4	27.5	18.3	36.6	7.5	3.5	11.6
Total	44.1	38.6	49.6	33.2	26.0	40.4	27.5	18.3	36.6	43.5	38.0	49.1

Male, Coloured

	Origin state											
	No disease			One disease			Multimorbidity			Weighted average		
Destination state	Est.	LCI	UCI	Est.	LCI	UCI	Est.	LCI	UCI	Est.	LCI	UCI
No disease	24.8	23.5	26.0	0.0	0.0	0.0	0.0	0.0	0.0	23.5	22.3	24.7
One disease	9.0	8.0	10.0	17.4	15.8	19.0	0.0	0.0	0.0	9.3	8.3	10.3
Multimorbidity	5.5	4.4	6.5	11.1	9.0	13.2	22.1	19.2	25.1	5.8	4.7	6.9
Total	39.2	37.7	40.8	28.5	26.4	30.6	22.1	19.2	25.1	38.6	37.1	40.2

Male, White

	Origin state											
	No disease			One disease			Multimorbidity			Weighted average		
Destination state	Est.	LCI	UCI	Est.	LCI	UCI	Est.	LCI	UCI	Est.	LCI	UCI
No disease	30.4	27.8	33.1	0.0	0.0	0.0	0.0	0.0	0.0	28.9	26.4	31.4
One disease	9.4	7.6	11.2	19.0	16.0	22.1	0.0	0.0	0.0	9.8	8.0	11.7
Multimorbidity	7.0	4.8	9.2	15.9	11.4	20.5	29.1	23.5	34.7	7.5	5.2	9.8
Total	46.8	44.1	49.6	35.0	30.9	39.1	29.1	23.5	34.7	46.2	43.4	49.0

Female, African

	Origin state											
	No disease			One disease			Multimorbidity			Weighted average		
Destination state	Est.	LCI	UCI	Est.	LCI	UCI	Est.	LCI	UCI	Est.	LCI	UCI
No disease	25.7	25.1	26.4	0.0	0.0	0.0	0.0	0.0	0.0	23.6	23.0	24.2
One disease	9.5	9.0	10.0	17.2	16.1	18.2	0.0	0.0	0.0	10.0	9.5	10.5
Multimorbidity	9.2	8.4	9.9	18.1	16.4	19.8	31.0	28.5	33.4	10.0	9.2	10.9
Total	44.4	43.5	45.3	35.3	33.7	36.8	31.0	28.5	33.4	43.6	42.7	44.5

Female, Asian/Indian

	Origin state											
	No disease			One disease			Multimorbidity			Weighted average		
Destination state	Est.	LCI	UCI	Est.	LCI	UCI	Est.	LCI	UCI	Est.	LCI	UCI
No disease	26.8	22.0	31.5	0.0	0.0	0.0	0	0.0	0.0	24.5	20.2	28.8
One disease	11.0	7.4	14.5	18.6	13.2	24.0	0	0.0	0.0	11.5	7.8	15.1
Multimorbidity	12.8	7.6	18.0	24.9	16.1	33.7	40	31.1	49.0	13.9	8.5	19.4
Total	50.5	45.8	55.2	43.5	36.7	50.4	40	31.1	49.0	49.9	45.1	54.7

Female, Coloured

	Origin state											
	No disease			One disease			Multimorbidity			Weighted average		
Destination state	Est.	LCI	UCI	Est.	LCI	UCI	Est.	LCI	UCI	Est.	LCI	UCI
No disease	24.4	23.1	25.6	0.0	0.0	0.0	0.0	0.0	0.0	22.3	21.2	23.5
One disease	10.8	9.8	11.9	18.3	16.6	20.0	0.0	0.0	0.0	11.3	10.2	12.4
Multimorbidity	11.0	9.4	12.6	20.4	17.6	23.2	34.4	31.0	37.7	11.9	10.2	13.6
Total	46.2	44.7	47.7	38.7	36.4	41.0	34.4	31.0	37.7	45.6	44.0	47.1

Female, White

	Origin state											
	No disease			One disease			Multimorbidity			Weighted average		
Destination state	Est.	LCI	UCI	Est.	LCI	UCI	Est.	LCI	UCI	Est.	LCI	UCI
No disease	29.6	26.9	32.3	0.0	0.0	0.0	0.0	0.0	0.0	27.1	24.7	29.6
One disease	10.7	8.8	12.7	19.2	15.9	22.5	0.0	0.0	0.0	11.3	9.3	13.3
Multimorbidity	12.2	9.4	15.0	25.9	20.7	31.0	41.6	36.4	46.8	13.5	10.5	16.5
Total	52.6	50.3	54.8	45.0	41.3	48.8	41.6	36.4	46.8	51.9	49.5	54.3

Model 3: Stratified by sex and education

Male, Less than secondary school

	Origin state											
	No disease			One disease			Multimorbidity			Weighted average		
Destination state	Est.	LCI	UCI	Est.	LCI	UCI	Est.	LCI	UCI	Est.	LCI	UCI
No disease	23.3	22.3	24.2	0.0	0.0	0.0	0.0	0.0	0.0	22.1	21.1	23.0
One disease	7.8	7.2	8.5	15.4	14.1	16.6	0.0	0.0	0.0	8.1	7.5	8.8
Multimorbidity	4.8	4.1	5.5	9.8	8.3	11.4	19.8	17.4	22.2	5.1	4.4	5.9
Total	35.9	34.8	37.0	25.2	23.6	26.8	19.8	17.4	22.2	35.4	34.3	36.5

Male, Secondary school

	Origin state											
	No disease			One disease			Multimorbidity			Weighted average		
Destination state	Est.	LCI	UCI	Est.	LCI	UCI	Est.	LCI	UCI	Est.	LCI	UCI
No disease	26.7	25.7	27.6	0.0	0.0	0.0	0.0	0.0	0.0	25.3	24.4	26.2
One disease	7.9	7.2	8.6	16.5	15.3	17.7	0.0	0.0	0.0	8.3	7.5	9.0
Multimorbidity	4.2	3.5	5.0	9.4	7.8	10.9	19.5	17.1	21.9	4.5	3.8	5.3
Total	38.8	37.6	40.0	25.9	24.3	27.5	19.5	17.1	21.9	38.1	36.9	39.3

Male, Post-secondary education

	Origin state											
	No disease			One disease			Multimorbidity			Weighted average		
Destination state	Est.	LCI	UCI	Est.	LCI	UCI	Est.	LCI	UCI	Est.	LCI	UCI
No disease	31.3	29.5	33.1	0.0	0.0	0.0	0.0	0.0	0.0	29.7	28.0	31.4
One disease	9.6	8.2	11.0	19.7	17.3	22.1	0.0	0.0	0.0	10.1	8.7	11.5
Multimorbidity	6.1	4.5	7.7	14.4	11.0	17.9	27.4	22.9	32.0	6.6	4.9	8.3
Total	47.0	44.8	49.2	34.1	30.8	37.4	27.4	22.9	32.0	46.3	44.1	48.5

Female, Less than secondary school

	Origin state											
	No disease			One disease			Multimorbidity			Weighted average		
Destination state	Est.	LCI	UCI	Est.	LCI	UCI	Est.	LCI	UCI	Est.	LCI	UCI
No disease	23.2	22.3	24.1	0.0	0.0	0.0	0.0	0.0	0.0	21.3	20.4	22.1
One disease	9.7	9.1	10.3	16.5	15.2	17.7	0.0	0.0	0.0	10.2	9.5	10.8
Multimorbidity	10.2	9.3	11.2	18.8	16.9	20.7	31.4	28.8	34.1	11.0	10.0	12.1
Total	43.2	42.1	44.2	35.3	33.6	37.0	31.4	28.8	34.1	42.5	41.4	43.5

Female, Secondary school

	Origin state											
	No disease			One disease			Multimorbidity			Weighted average		
Destination state	Est.	LCI	UCI	Est.	LCI	UCI	Est.	LCI	UCI	Est.	LCI	UCI
No disease	26.6	25.7	27.5	0.0	0.0	0.0	0.0	0.0	0.0	24.4	23.5	25.2
One disease	9.8	9.1	10.6	17.8	16.5	19.1	0.0	0.0	0.0	10.4	9.6	11.2
Multimorbidity	8.9	7.8	10.1	18.0	15.8	20.1	31.1	28.2	33.9	9.8	8.6	11.0
Total	45.4	44.1	46.6	35.8	33.8	37.7	31.1	28.2	33.9	44.5	43.3	45.8

Female, Post-secondary education

	Origin state											
	No disease			One disease			Multimorbidity			Weighted average		
Destination state	Est.	LCI	UCI	Est.	LCI	UCI	Est.	LCI	UCI	Est.	LCI	UCI
No disease	30.5	28.7	32.2	0.0	0.0	0.0	0.0	0.0	0.0	27.9	26.3	29.5
One disease	11.1	9.6	12.6	20.1	17.5	22.7	0.0	0.0	0.0	11.7	10.2	13.2
Multimorbidity	10.9	8.8	12.9	23.9	19.9	28.0	39.8	35.4	44.3	12.1	9.9	14.3
Total	52.4	50.6	54.3	44.0	40.9	47.2	39.8	35.4	44.3	51.7	49.7	53.7

Model 4: Stratified by sex and race*binary education interaction

Male, African, Less than secondary school

	Origin state											
	No disease			One disease			Multimorbidity			Weighted average		
Destination state	Est.	LCI	UCI	Est.	LCI	UCI	Est.	LCI	UCI	Est.	LCI	UCI
No disease	23.7	22.7	24.7	0.0	0.0	0.0	0	0.0	0.0	22.5	21.5	23.5
One disease	7.7	7.0	8.4	15.3	14.0	16.6	0	0.0	0.0	8.0	7.3	8.7
Multimorbidity	4.8	4.0	5.5	9.9	8.3	11.6	20	17.5	22.5	5.1	4.3	5.8
Total	36.2	35.0	37.3	25.2	23.6	26.9	20	17.5	22.5	35.6	34.4	36.7

Male, African, Secondary school or more

	Origin state											
	No disease			One disease			Multimorbidity			Weighted average		
Destination state	Est.	LCI	UCI	Est.	LCI	UCI	Est.	LCI	UCI	Est.	LCI	UCI
No disease	27.1	26.1	28.1	0.0	0.0	0.0	0.0	0.0	0.0	25.7	24.8	26.7
One disease	7.5	6.8	8.2	16.3	15.1	17.5	0.0	0.0	0.0	7.9	7.2	8.6
Multimorbidity	3.7	3.0	4.4	8.6	7.1	10.1	18.4	16.1	20.7	4.0	3.3	4.7
Total	38.4	37.1	39.6	24.9	23.3	26.5	18.4	16.1	20.7	37.6	36.4	38.9

Male, Coloured, Less than secondary school

	Origin state											
	No disease			One disease			Multimorbidity			Weighted average		
Destination state	Est.	LCI	UCI	Est.	LCI	UCI	Est.	LCI	UCI	Est.	LCI	UCI
No disease	21.6	20.1	23.2	0.0	0.0	0.0	0.0	0.0	0.0	20.5	19.1	22.0
One disease	8.6	7.5	9.8	16.0	14.2	17.8	0.0	0.0	0.0	8.9	7.8	10.1
Multimorbidity	5.3	4.1	6.6	10.3	8.0	12.6	20.7	17.4	23.9	5.6	4.3	6.9
Total	35.6	33.7	37.4	26.3	24.0	28.6	20.7	17.4	23.9	35.1	33.2	36.9

Male, Coloured, Secondary school or more

	Origin state											
	No disease			One disease			Multimorbidity			Weighted average		
Destination state	Est.	LCI	UCI	Est.	LCI	UCI	Est.	LCI	UCI	Est.	LCI	UCI
No disease	28.3	26.2	30.4	0.0	0.0	0.0	0.0	0.0	0.0	26.8	24.8	28.8
One disease	10.6	8.7	12.4	20.0	17.0	23.0	0.0	0.0	0.0	11.0	9.1	12.9
Multimorbidity	6.9	4.8	9.0	14.9	10.6	19.1	28.2	22.8	33.6	7.4	5.1	9.6
Total	45.8	42.9	48.7	34.8	30.8	38.9	28.2	22.8	33.6	45.2	42.2	48.1

Male, White, Less than secondary school

	Origin state											
	No disease			One disease			Multimorbidity			Weighted average		
Destination state	Est.	LCI	UCI	Est.	LCI	UCI	Est.	LCI	UCI	Est.	LCI	UCI
No disease	22.2	13.6	30.8	0.0	0.0	0.0	0.0	0.0	0.0	21.1	12.9	29.2
One disease	11.4	4.5	18.3	18.6	9.3	27.9	0.0	0.0	0.0	11.7	4.8	18.7
Multimorbidity	9.9	0.5	19.3	17.6	2.6	32.6	31.1	13.7	48.5	10.3	0.6	20.0
Total	43.5	32.9	54.1	36.2	22.7	49.7	31.1	13.7	48.5	43.1	32.4	53.8

Male, White, Secondary school or more

	Origin state											
	No disease			One disease			Multimorbidity			Weighted average		
Destination state	Est.	LCI	UCI	Est.	LCI	UCI	Est.	LCI	UCI	Est.	LCI	UCI
No disease	29.9	27.1	32.7	0.0	0.0	0.0	0.0	0.0	0	28.4	25.7	31.0
One disease	9.3	7.5	11.2	18.5	15.3	21.6	0.0	0.0	0	9.7	7.8	11.6
Multimorbidity	7.1	4.8	9.4	16.1	11.3	20.9	29.1	23.3	35	7.6	5.2	10.0
Total	46.4	43.4	49.3	34.6	30.3	38.9	29.1	23.3	35	45.7	42.8	48.7

Female, African, Less than secondary school

	Origin state											
	No disease			One disease			Multimorbidity			Weighted average		
Destination state	Est.	LCI	UCI	Est.	LCI	UCI	Est.	LCI	UCI	Est.	LCI	UCI
No disease	23.7	22.7	24.7	0.0	0.0	0.0	0.0	0	0.0	21.7	20.8	22.6
One disease	9.6	8.9	10.3	16.4	15.0	17.7	0.0	0	0.0	10.0	9.3	10.7
Multimorbidity	10.1	9.1	11.1	19.0	17.0	21.1	31.7	29	34.4	11.0	9.9	12.1
Total	43.4	42.3	44.5	35.4	33.6	37.2	31.7	29	34.4	42.7	41.5	43.8

Female, African, Secondary school or more

	Origin state											
	No disease			One disease			Multimorbidity			Weighted average		
Destination state	Est.	LCI	UCI	Est.	LCI	UCI	Est.	LCI	UCI	Est.	LCI	UCI
No disease	27.2	26.2	28.1	0.0	0.0	0.0	0.0	0	0.0	24.9	24.0	25.7
One disease	9.6	8.8	10.4	17.7	16.4	19.0	0.0	0	0.0	10.1	9.3	10.9
Multimorbidity	8.1	7.0	9.2	16.9	14.8	19.0	29.8	27	32.6	8.9	7.7	10.1
Total	44.8	43.6	46.1	34.6	32.7	36.5	29.8	27	32.6	43.9	42.7	45.2

Female, Coloured, Less than secondary school

	Origin state											
	No disease			One disease			Multimorbidity			Weighted average		
Destination state	Est.	LCI	UCI	Est.	LCI	UCI	Est.	LCI	UCI	Est.	LCI	UCI
No disease	21.4	19.9	23.0	0.0	0.0	0.0	0.0	0.0	0.0	19.6	18.2	21.0
One disease	10.6	9.3	11.9	17.0	15.1	19.0	0.0	0.0	0.0	11.0	9.7	12.3
Multimorbidity	11.1	9.2	13.0	19.4	16.2	22.6	32.6	28.8	36.3	11.9	9.9	13.9
Total	43.1	41.2	45.0	36.5	33.9	39.1	32.6	28.8	36.3	42.5	40.6	44.4

Female, Coloured, Secondary school or more

	Origin state											
	No disease			One disease			Multimorbidity			Weighted average		
Destination state	Est.	LCI	UCI	Est.	LCI	UCI	Est.	LCI	UCI	Est.	LCI	UCI
No disease	27.4	25.3	29.5	0.0	0.0	0.0	0.0	0.0	0.0	25.1	23.2	27.0
One disease	12.1	10.1	14.1	20.3	17.1	23.5	0.0	0.0	0.0	12.6	10.6	14.7
Multimorbidity	12.3	9.5	15.0	24.5	19.5	29.4	40.7	35.5	45.9	13.4	10.5	16.3
Total	51.8	49.3	54.2	44.7	40.9	48.5	40.7	35.5	45.9	51.1	48.6	53.7

Female, White, Less than secondary school

	Origin state											
	No disease			One disease			Multimorbidity			Weighted average		
Destination state	Est.	LCI	UCI	Est.	LCI	UCI	Est.	LCI	UCI	Est.	LCI	UCI
No disease	21.3	12.6	29.9	0.0	0.0	0.0	0.0	0	0.0	19.5	11.6	27.4
One disease	12.6	5.2	20.0	18.5	8.6	28.5	0.0	0	0.0	13.0	5.4	20.5
Multimorbidity	16.7	5.3	28.1	27.7	11.5	43.9	43.4	28	58.7	17.7	6.0	29.5
Total	50.6	41.7	59.4	46.2	34.4	58.1	43.4	28	58.7	50.2	41.1	59.2

Female, White, Secondary school or more

	Origin state											
	No disease			One disease			Multimorbidity			Weighted average		
Destination state	Est.	LCI	UCI	Est.	LCI	UCI	Est.	LCI	UCI	Est.	LCI	UCI
No disease	29.1	26.3	31.9	0.0	0.0	0.0	0.0	0.0	0	26.7	24.0	29.3
One disease	10.6	8.6	12.7	18.6	15.2	22.1	0.0	0.0	0	11.2	9.0	13.3
Multimorbidity	12.5	9.4	15.5	26.1	20.6	31.6	41.6	36.1	47	13.7	10.5	16.9
Total	52.2	49.8	54.7	44.7	40.8	48.7	41.6	36.1	47	51.6	49.0	54.1

2 Exclude tuberculosis from multimorbidity definition

2.1 Origin disease state distribution

Origin disease state	Male (n=7224)	Female (n=10,806)	Overall (N=18,030)
No disease	5,784 (80.1%)	7,341 (67.9%)	13,125 (72.8%)
One disease	1,146 (15.9%)	2,566 (23.7%)	3,712 (20.6%)
Multimorbidity	294 (4.1%)	899 (8.3%)	1,193 (6.6%)

2.2 Expectancy estimates

Model 1: Stratified by sex

Male

	Origin state											
	No disease			One disease			Multimorbidity			Weighted average		
Destination state	Est.	LCI	UCI	Est.	LCI	UCI	Est.	LCI	UCI	Est.	LCI	UCI
No disease	21.7	21.1	22.4	0.0	0.0	0.0	0.0	0.0	0.0	20.4	19.8	21.0
One disease	9.5	9.0	10.0	16.6	15.6	17.5	0.0	0.0	0.0	9.9	9.3	10.4
Multimorbidity	7.4	6.7	8.0	12.4	11.1	13.7	21.3	19.3	23.2	7.7	7.0	8.4
Total	38.6	37.8	39.5	29.0	27.7	30.3	21.3	19.3	23.2	38.0	37.1	38.9

Female

	Origin state											
	No disease			One disease			Multimorbidity			Weighted average		
Destination state	Est.	LCI	UCI	Est.	LCI	UCI	Est.	LCI	UCI	Est.	LCI	UCI
No disease	19.7	19.2	20.2	0.0	0.0	0.0	0.0	0.0	0.0	17.7	17.2	18.1
One disease	11.2	10.7	11.7	17.0	16.2	17.9	0.0	0.0	0.0	11.6	11.1	12.1
Multimorbidity	14.5	13.7	15.3	22.2	20.8	23.6	33.7	31.8	35.7	15.4	14.6	16.2
Total	45.4	44.6	46.2	39.2	38.0	40.5	33.7	31.8	35.7	44.7	43.9	45.6

Model 2: Stratified by sex and race

Male, African

	Origin state											
	No disease			One disease			Multimorbidity			Weighted average		
Destination state	Est.	LCI	UCI	Est.	LCI	UCI	Est.	LCI	UCI	Est.	LCI	UCI
No disease	22.0	21.4	22.7	0.0	0.0	0.0	0.0	0.0	0.0	20.7	20.1	21.3
One disease	9.1	8.6	9.7	16.3	15.3	17.3	0.0	0.0	0.0	9.5	8.9	10.1
Multimorbidity	6.6	5.9	7.3	11.3	10.0	12.6	19.8	17.8	21.7	6.9	6.2	7.6
Total	37.8	36.9	38.7	27.6	26.2	28.9	19.8	17.8	21.7	37.1	36.2	38.0

Male, Asian/Indian

	Origin state											
	No disease			One disease			Multimorbidity			Weighted average		
Destination state	Est.	LCI	UCI	Est.	LCI	UCI	Est.	LCI	UCI	Est.	LCI	UCI
No disease	25.3	21.2	29.4	0.0	0.0	0.0	0.0	0.0	0.0	23.8	19.9	27.6
One disease	7.8	5.2	10.4	13.9	10.2	17.7	0.0	0.0	0.0	8.1	5.5	10.8
Multimorbidity	11.8	7.1	16.5	21.3	13.6	28.9	31.1	22.4	39.7	12.4	7.6	17.2
Total	44.9	39.3	50.5	35.2	27.8	42.6	31.1	22.4	39.7	44.3	38.6	49.9

Male, Coloured

	Origin state											
	No disease			One disease			Multimorbidity			Weighted average		
Destination state	Est.	LCI	UCI	Est.	LCI	UCI	Est.	LCI	UCI	Est.	LCI	UCI
No disease	19.6	18.6	20.6	0.0	0.0	0.0	0.0	0.0	0.0	18.4	17.5	19.3
One disease	10.9	9.9	11.9	17.6	16.2	19.0	0.0	0.0	0.0	11.3	10.3	12.3
Multimorbidity	8.9	7.6	10.1	13.9	11.9	15.9	23.5	20.9	26.2	9.2	7.9	10.5
Total	39.4	37.8	41.0	31.5	29.5	33.5	23.5	20.9	26.2	38.9	37.3	40.5

Male, White

	Origin state											
	No disease			One disease			Multimorbidity			Weighted average		
Destination state	Est.	LCI	UCI	Est.	LCI	UCI	Est.	LCI	UCI	Est.	LCI	UCI
No disease	25.5	23.2	27.8	0.0	0.0	0.0	0.0	0.0	0.0	24.0	21.8	26.1
One disease	10.4	8.8	12.1	17.8	15.4	20.2	0.0	0.0	0.0	10.8	9.2	12.5
Multimorbidity	11.6	9.2	14.0	20.6	16.5	24.8	32.1	27.1	37.1	12.2	9.7	14.7
Total	47.6	44.8	50.3	38.4	34.5	42.3	32.1	27.1	37.1	47.0	44.2	49.8

Female, African

	Origin state											
	No disease			One disease			Multimorbidity			Weighted average		
Destination state	Est.	LCI	UCI	Est.	LCI	UCI	Est.	LCI	UCI	Est.	LCI	UCI
No disease	20.0	19.4	20.5	0.0	0.0	0.0	0.0	0.0	0.0	17.9	17.5	18.4
One disease	11.0	10.5	11.5	16.9	16.0	17.8	0.0	0.0	0.0	11.4	10.9	11.9
Multimorbidity	13.8	13.0	14.6	21.3	19.8	22.7	32.4	30.4	34.5	14.6	13.8	15.5
Total	44.7	43.9	45.6	38.1	36.8	39.5	32.4	30.4	34.5	44.0	43.1	44.9

Female, Asian/Indian

	Origin state											
	No disease			One disease			Multimorbidity			Weighted average		
Destination state	Est.	LCI	UCI	Est.	LCI	UCI	Est.	LCI	UCI	Est.	LCI	UCI
No disease	22.7	18.8	26.6	0.0	0.0	0.0	0.0	0.0	0.0	20.4	16.9	23.9
One disease	8.6	5.8	11.4	13.5	9.8	17.3	0.0	0.0	0.0	9.0	6.2	11.8
Multimorbidity	20.1	14.9	25.3	32.7	25.4	40.1	44.0	36.6	51.4	21.5	16.1	26.9
Total	51.4	46.9	56.0	46.3	40.0	52.6	44.0	36.6	51.4	50.9	46.2	55.6

Female, Coloured

	Origin state											
	No disease			One disease			Multimorbidity			Weighted average		
Destination state	Est.	LCI	UCI	Est.	LCI	UCI	Est.	LCI	UCI	Est.	LCI	UCI
No disease	17.4	16.5	18.3	0.0	0.0	0.0	0.0	0.0	0.0	15.6	14.8	16.4
One disease	12.5	11.4	13.5	17.8	16.4	19.2	0.0	0.0	0.0	12.9	11.8	13.9
Multimorbidity	16.9	15.3	18.5	24.3	22.0	26.6	36.8	34.0	39.5	17.8	16.1	19.5
Total	46.8	45.3	48.3	42.1	40.2	44.1	36.8	34.0	39.5	46.3	44.7	47.8

Female, White

	Origin state											
	No disease			One disease			Multimorbidity			Weighted average		
Destination state	Est.	LCI	UCI	Est.	LCI	UCI	Est.	LCI	UCI	Est.	LCI	UCI
No disease	22.7	20.6	24.9	0.0	0.0	0.0	0.0	0.0	0.0	20.4	18.4	22.3
One disease	11.4	9.7	13.2	17.3	14.8	19.7	0.0	0.0	0.0	11.9	10.1	13.6
Multimorbidity	19.2	16.5	22.0	31.2	27.2	35.2	44.9	40.7	49.1	20.6	17.7	23.4
Total	53.4	51.2	55.6	48.5	45.3	51.6	44.9	40.7	49.1	52.8	50.5	55.1

Model 3: Stratified by sex and education

Male, Less than secondary school

	Origin state											
	No disease			One disease			Multimorbidity			Weighted average		
Destination state	Est.	LCI	UCI	Est.	LCI	UCI	Est.	LCI	UCI	Est.	LCI	UCI
No disease	20.0	19.2	20.8	0.0	0.0	0.0	0	0.0	0.0	18.7	18.0	19.5
One disease	9.1	8.5	9.7	15.5	14.3	16.6	0	0.0	0.0	9.4	8.8	10.1
Multimorbidity	7.2	6.4	8.0	11.7	10.3	13.2	20	17.9	22.2	7.5	6.7	8.4
Total	36.3	35.2	37.4	27.2	25.7	28.7	20	17.9	22.2	35.7	34.6	36.8

Male, Secondary school

	Origin state											
	No disease			One disease			Multimorbidity			Weighted average		
Destination state	Est.	LCI	UCI	Est.	LCI	UCI	Est.	LCI	UCI	Est.	LCI	UCI
No disease	22.3	21.6	23.1	0.0	0.0	0.0	0.0	0.0	0.0	21.0	20.2	21.7
One disease	9.5	8.7	10.2	16.5	15.4	17.6	0.0	0.0	0.0	9.8	9.1	10.6
Multimorbidity	7.1	6.2	8.0	12.1	10.5	13.6	20.7	18.5	22.9	7.4	6.5	8.4
Total	38.9	37.6	40.1	28.5	26.9	30.2	20.7	18.5	22.9	38.2	37.0	39.5

Male, Post-secondary education

	Origin state											
	No disease			One disease			Multimorbidity			Weighted average		
Destination state	Est.	LCI	UCI	Est.	LCI	UCI	Est.	LCI	UCI	Est.	LCI	UCI
No disease	24.9	23.5	26.3	0.0	0.0	0.0	0.0	0.0	0.0	23.3	22.0	24.7
One disease	12.4	11.0	13.8	20.4	18.3	22.4	0.0	0.0	0.0	12.8	11.4	14.3
Multimorbidity	10.4	8.5	12.3	18.1	14.9	21.3	30.1	25.9	34.3	10.9	9.0	12.9
Total	47.7	45.5	49.9	38.5	35.4	41.6	30.1	25.9	34.3	47.1	44.8	49.4

Female, Less than secondary school

	Origin state											
	No disease			One disease			Multimorbidity			Weighted average		
Destination state	Est.	LCI	UCI	Est.	LCI	UCI	Est.	LCI	UCI	Est.	LCI	UCI
No disease	18.0	17.3	18.8	0.0	0.0	0.0	0.0	0.0	0.0	16.2	15.5	16.9
One disease	10.9	10.3	11.5	16.1	15.0	17.2	0.0	0.0	0.0	11.3	10.6	11.9
Multimorbidity	14.7	13.7	15.7	21.7	20.1	23.4	32.6	30.3	34.8	15.5	14.5	16.6
Total	43.6	42.6	44.7	37.8	36.4	39.3	32.6	30.3	34.8	43.0	41.9	44.1

Female, Secondary school

	Origin state											
	No disease			One disease			Multimorbidity			Weighted average		
Destination state	Est.	LCI	UCI	Est.	LCI	UCI	Est.	LCI	UCI	Est.	LCI	UCI
No disease	20.2	19.5	20.8	0.0	0.0	0.0	0.0	0	0.0	18.1	17.5	18.7
One disease	11.3	10.6	12.0	17.1	16.0	18.2	0.0	0	0.0	11.7	11.0	12.5
Multimorbidity	14.2	13.0	15.5	22.0	20.1	23.9	33.4	31	35.8	15.1	13.8	16.4
Total	45.7	44.5	46.9	39.1	37.4	40.8	33.4	31	35.8	45.0	43.7	46.2

Female, Post-secondary education

	Origin state											
	No disease			One disease			Multimorbidity			Weighted average		
Destination state	Est.	LCI	UCI	Est.	LCI	UCI	Est.	LCI	UCI	Est.	LCI	UCI
No disease	22.0	20.8	23.3	0.0	0.0	0.0	0.0	0.0	0.0	19.8	18.6	20.9
One disease	13.8	12.3	15.3	20.2	18.1	22.3	0.0	0.0	0.0	14.3	12.8	15.8
Multimorbidity	17.4	15.3	19.5	27.9	24.7	31.1	42.9	39.3	46.6	18.6	16.4	20.8
Total	53.2	51.4	55.0	48.1	45.5	50.7	42.9	39.3	46.6	52.6	50.7	54.5

Model 4: Stratified by sex and race*binary education interaction

Male, African, Less than secondary school

	Origin state											
	No disease			One disease			Multimorbidity			Weighted average		
Destination state	Est.	LCI	UCI	Est.	LCI	UCI	Est.	LCI	UCI	Est.	LCI	UCI
No disease	20.3	19.4	21.2	0.0	0.0	0.0	0.0	0.0	0.0	19.1	18.3	19.9
One disease	9.1	8.4	9.8	15.5	14.3	16.7	0.0	0.0	0.0	9.4	8.7	10.1
Multimorbidity	7.1	6.2	7.9	11.7	10.2	13.3	20.1	17.9	22.4	7.4	6.5	8.2
Total	36.4	35.3	37.6	27.2	25.6	28.8	20.1	17.9	22.4	35.9	34.7	37.0

Male, African, Secondary school or more

	Origin state											
	No disease			One disease			Multimorbidity			Weighted average		
Destination state	Est.	LCI	UCI	Est.	LCI	UCI	Est.	LCI	UCI	Est.	LCI	UCI
No disease	23.1	22.3	23.9	0.0	0.0	0.0	0.0	0.0	0.0	21.7	20.9	22.4
One disease	9.3	8.6	10.1	16.7	15.5	17.8	0.0	0.0	0.0	9.7	8.9	10.5
Multimorbidity	6.0	5.2	6.9	10.7	9.3	12.2	19.2	17.1	21.3	6.4	5.5	7.3
Total	38.5	37.2	39.7	27.4	25.8	29.0	19.2	17.1	21.3	37.8	36.5	39.0

Male, Coloured, Less than secondary school

	Origin state											
	No disease			One disease			Multimorbidity			Weighted average		
Destination state	Est.	LCI	UCI	Est.	LCI	UCI	Est.	LCI	UCI	Est.	LCI	UCI
No disease	18.5	17.2	19.8	0.0	0.0	0.0	0	0.0	0.0	17.4	16.1	18.6
One disease	9.7	8.6	10.9	15.8	14.2	17.4	0	0.0	0.0	10.0	8.9	11.2
Multimorbidity	7.8	6.4	9.2	12.4	10.2	14.5	21	18.2	23.8	8.1	6.7	9.5
Total	36.0	34.2	37.9	28.2	25.9	30.4	21	18.2	23.8	35.5	33.6	37.4

Male, Coloured, Secondary school or more

	Origin state											
	No disease			One disease			Multimorbidity			Weighted average		
Destination state	Est.	LCI	UCI	Est.	LCI	UCI	Est.	LCI	UCI	Est.	LCI	UCI
No disease	20.1	18.8	21.5	0.0	0.0	0.0	0	0.0	0.0	18.9	17.6	20.2
One disease	13.4	11.6	15.2	19.9	17.5	22.3	0	0.0	0.0	13.7	11.9	15.5
Multimorbidity	12.7	10.0	15.4	19.6	15.6	23.7	32	26.9	37.1	13.1	10.4	15.9
Total	46.2	43.2	49.2	39.6	35.7	43.4	32	26.9	37.1	45.8	42.7	48.8

Male, White, Less than secondary school

	Origin state											
	No disease			One disease			Multimorbidity			Weighted average		
Destination state	Est.	LCI	UCI	Est.	LCI	UCI	Est.	LCI	UCI	Est.	LCI	UCI
No disease	22.7	13.4	32.0	0.0	0.0	0.0	0.0	0.0	0.0	21.3	12.6	30.0
One disease	5.7	2.2	9.2	9.8	5.1	14.5	0.0	0.0	0.0	5.9	2.3	9.5
Multimorbidity	16.7	6.2	27.2	28.5	13.7	43.3	37.1	21.9	52.3	17.5	6.8	28.2
Total	45.1	34.2	56.0	38.3	24.2	52.4	37.1	21.9	52.3	44.7	33.6	55.8

Male, White, Secondary school or more

	Origin state											
	No disease			One disease			Multimorbidity			Weighted average		
Destination state	Est.	LCI	UCI	Est.	LCI	UCI	Est.	LCI	UCI	Est.	LCI	UCI
No disease	24.9	22.6	27.3	0.0	0.0	0.0	0.0	0.0	0.0	23.4	21.2	25.6
One disease	10.8	9.0	12.6	17.9	15.3	20.5	0.0	0.0	0.0	11.2	9.4	12.9
Multimorbidity	11.4	8.9	14.0	20.2	15.9	24.5	31.6	26.4	36.9	12.0	9.4	14.7
Total	47.2	44.2	50.1	38.1	34.0	42.1	31.6	26.4	36.9	46.6	43.6	49.6

Female, African, Less than secondary school

	Origin state											
	No disease			One disease			Multimorbidity			Weighted average		
Destination state	Est.	LCI	UCI	Est.	LCI	UCI	Est.	LCI	UCI	Est.	LCI	UCI
No disease	18.3	17.5	19.1	0.0	0.0	0.0	0.0	0.0	0.0	16.4	15.7	17.1
One disease	10.9	10.2	11.5	16.1	14.9	17.2	0.0	0.0	0.0	11.2	10.5	11.9
Multimorbidity	14.7	13.6	15.7	21.9	20.2	23.7	32.9	30.6	35.2	15.5	14.4	16.6
Total	43.8	42.7	44.9	38.0	36.4	39.5	32.9	30.6	35.2	43.2	42.0	44.3

Female, African, Secondary school or more

	Origin state											
	No disease			One disease			Multimorbidity			Weighted average		
Destination state	Est.	LCI	UCI	Est.	LCI	UCI	Est.	LCI	UCI	Est.	LCI	UCI
No disease	20.9	20.3	21.6	0.0	0.0	0.0	0.0	0.0	0.0	18.8	18.2	19.4
One disease	11.3	10.6	12.1	17.4	16.3	18.5	0.0	0.0	0.0	11.8	11.0	12.6
Multimorbidity	12.8	11.6	14.0	20.4	18.5	22.3	31.7	29.2	34.1	13.7	12.4	15.0
Total	45.1	43.8	46.3	37.8	36.1	39.5	31.7	29.2	34.1	44.3	43.0	45.6

Female, Coloured, Less than secondary school

	Origin state											
	No disease			One disease			Multimorbidity			Weighted average		
Destination state	Est.	LCI	UCI	Est.	LCI	UCI	Est.	LCI	UCI	Est.	LCI	UCI
No disease	16.5	15.3	17.8	0.0	0.0	0.0	0.0	0.0	0	14.8	13.7	16.0
One disease	11.4	10.2	12.6	16.3	14.6	17.9	0.0	0.0	0	11.7	10.5	13.0
Multimorbidity	15.8	13.9	17.7	22.7	20.0	25.3	33.9	30.7	37	16.6	14.7	18.6
Total	43.8	41.9	45.6	38.9	36.6	41.3	33.9	30.7	37	43.2	41.3	45.1

Female, Coloured, Secondary school or more

	Origin state											
	No disease			One disease			Multimorbidity			Weighted average		
Destination state	Est.	LCI	UCI	Est.	LCI	UCI	Est.	LCI	UCI	Est.	LCI	UCI
No disease	17.5	16.3	18.8	0.0	0.0	0.0	0.0	0.0	0	15.7	14.6	16.9
One disease	14.4	12.5	16.2	19.5	17.0	21.9	0.0	0.0	0	14.7	12.8	16.6
Multimorbidity	20.7	17.8	23.6	29.7	25.7	33.6	44.8	40.5	49	21.8	18.8	24.7
Total	52.6	50.2	55.0	49.1	46.0	52.3	44.8	40.5	49	52.2	49.7	54.7

Female, White, Less than secondary school

	Origin state											
	No disease			One disease			Multimorbidity			Weighted average		
Destination state	Est.	LCI	UCI	Est.	LCI	UCI	Est.	LCI	UCI	Est.	LCI	UCI
No disease	20.1	11.3	28.9	0.0	0.0	0.0	0.0	0.0	0	18.0	10.2	25.9
One disease	6.1	2.5	9.7	9.3	4.8	13.8	0.0	0.0	0	6.3	2.7	9.9
Multimorbidity	26.1	15.5	36.7	39.9	27.9	51.9	48.8	37.5	60	27.6	17.0	38.2
Total	52.2	44.0	60.5	49.2	38.6	59.7	48.8	37.5	60	51.9	43.4	60.4

Female, White, Secondary school or more

	Origin state											
	No disease			One disease			Multimorbidity			Weighted average		
Destination state	Est.	LCI	UCI	Est.	LCI	UCI	Est.	LCI	UCI	Est.	LCI	UCI
No disease	22.1	19.8	24.3	0.0	0.0	0.0	0.0	0	0.0	19.8	17.8	21.8
One disease	11.9	10.0	13.7	17.5	14.8	20.1	0.0	0	0.0	12.3	10.3	14.2
Multimorbidity	19.1	16.2	22.0	30.7	26.4	35.0	44.4	40	48.9	20.4	17.4	23.5
Total	53.0	50.7	55.4	48.1	44.8	51.5	44.4	40	48.9	52.5	50.0	54.9

3 Exclude hypertension and tuberculosis from multimorbidity definition

3.1 Origin disease state distribution

Origin disease state	Male (n=7,224)	Female (n=10,806)	Overall (N=18,030)
No disease	6,543 (90.6%)	9,171 (84.9%)	15,714 (87.2%)
One disease	608 (8.4%)	1,405 (13.0%)	2,013 (11.2%)
Multimorbidity	73 (1.0%)	230 (2.1%)	303 (1.7%)

3.2 Expectancy estimates

Model 1: Stratified by sex

Male

	Origin state											
	No disease			One disease			Multimorbidity			Weighted average		
Destination state	Est.	LCI	UCI	Est.	LCI	UCI	Est.	LCI	UCI	Est.	LCI	UCI
No disease	29.7	28.9	30.4	0.0	0.0	0.0	0.0	0.0	0.0	28.6	27.8	29.4
One disease	6.6	6.1	7.1	16.9	15.6	18.1	0.0	0.0	0.0	6.9	6.4	7.4
Multimorbidity	2.7	2.3	3.1	7.0	5.7	8.3	16.4	14.0	18.8	2.9	2.4	3.3
Total	38.9	38.1	39.8	23.9	22.5	25.4	16.4	14.0	18.8	38.4	37.5	39.2

Female

	Origin state											
	No disease			One disease			Multimorbidity			Weighted average		
Destination state	Est.	LCI	UCI	Est.	LCI	UCI	Est.	LCI	UCI	Est.	LCI	UCI
No disease	28.5	27.8	29.1	0.0	0.0	0.0	0.0	0.0	0.0	26.8	26.2	27.5
One disease	9.7	9.1	10.2	19.3	18.0	20.6	0.0	0.0	0.0	10.1	9.6	10.7
Multimorbidity	7.4	6.7	8.0	16.3	14.5	18.0	29.8	26.9	32.8	7.9	7.2	8.7
Total	45.5	44.7	46.3	35.6	33.9	37.2	29.8	26.9	32.8	44.9	44.0	45.7

Model 2: Stratified by sex and race

Male, African

	Origin state											
	No disease			One disease			Multimorbidity			Weighted average		
Destination state	Est.	LCI	UCI	Est.	LCI	UCI	Est.	LCI	UCI	Est.	LCI	UCI
No disease	29.4	28.6	30.2	0.0	0.0	0.0	0.0	0.0	0.0	28.4	27.6	29.2
One disease	6.4	5.8	6.9	16.7	15.4	17.9	0.0	0.0	0.0	6.7	6.1	7.2
Multimorbidity	2.3	1.9	2.7	6.0	4.8	7.2	14.9	12.6	17.2	2.4	2.0	2.9
Total	38.1	37.2	38.9	22.7	21.3	24.1	14.9	12.6	17.2	37.5	36.6	38.4

Male, Asian/Indian

	Origin state											
	No disease			One disease			Multimorbidity			Weighted average		
Destination state	Est.	LCI	UCI	Est.	LCI	UCI	Est.	LCI	UCI	Est.	LCI	UCI
No disease	29.3	24.6	33.9	0.0	0.0	0.0	0.0	0.0	0.0	28.2	23.7	32.7
One disease	9.1	5.7	12.4	19.1	13.7	24.5	0.0	0.0	0.0	9.4	6.0	12.7
Multimorbidity	5.9	2.4	9.4	13.7	6.1	21.2	26.4	16.6	36.1	6.2	2.5	9.9
Total	44.2	38.6	49.9	32.8	25.3	40.3	26.4	16.6	36.1	43.8	38.2	49.5

Male, Coloured

	Origin state											
	No disease			One disease			Multimorbidity			Weighted average		
Destination state	Est.	LCI	UCI	Est.	LCI	UCI	Est.	LCI	UCI	Est.	LCI	UCI
No disease	30.0	28.6	31.4	0.0	0.0	0.0	0.0	0.0	0.0	29.0	27.6	30.3
One disease	6.7	5.8	7.6	16.8	15.1	18.6	0.0	0.0	0.0	7.0	6.2	7.9
Multimorbidity	3.2	2.4	4.0	8.3	6.3	10.4	18.3	15.1	21.6	3.4	2.6	4.2
Total	40.0	38.4	41.5	25.2	23.0	27.3	18.3	15.1	21.6	39.4	37.9	41.0

Male, White

	Origin state											
	No disease			One disease			Multimorbidity			Weighted average		
Destination state	Est.	LCI	UCI	Est.	LCI	UCI	Est.	LCI	UCI	Est.	LCI	UCI
No disease	32.6	30.0	35.1	0.0	0.0	0.0	0.0	0.0	0.0	31.4	28.9	33.9
One disease	8.6	6.9	10.4	19.3	16.1	22.5	0.0	0.0	0.0	9.0	7.2	10.7
Multimorbidity	6.1	4.1	8.1	15.6	10.8	20.4	29.0	22.8	35.2	6.5	4.4	8.6
Total	47.3	44.6	50.1	34.9	30.5	39.2	29.0	22.8	35.2	46.9	44.1	49.7

Female, African

	Origin state											
	No disease			One disease			Multimorbidity			Weighted average		
Destination state	Est.	LCI	UCI	Est.	LCI	UCI	Est.	LCI	UCI	Est.	LCI	UCI
No disease	28.4	27.7	29.1	0.0	0.0	0.0	0.0	0.0	0.0	26.7	26.1	27.4
One disease	9.6	9.0	10.1	19.3	18.0	20.6	0.0	0.0	0.0	10.1	9.5	10.7
Multimorbidity	6.8	6.1	7.5	15.1	13.3	16.9	28.4	25.4	31.3	7.3	6.6	8.1
Total	44.8	43.9	45.6	34.4	32.8	36.1	28.4	25.4	31.3	44.2	43.3	45.0

Female, Asian/Indian

	Origin state											
	No disease			One disease			Multimorbidity			Weighted average		
Destination state	Est.	LCI	UCI	Est.	LCI	UCI	Est.	LCI	UCI	Est.	LCI	UCI
No disease	26.8	22.2	31.5	0.0	0.0	0.0	0.0	0.0	0.0	25.3	21.0	29.6
One disease	11.5	7.6	15.4	19.7	13.8	25.7	0.0	0.0	0.0	11.9	8.0	15.9
Multimorbidity	12.9	7.7	18.1	25.7	16.6	34.8	42.0	32.9	51.1	13.7	8.3	19.1
Total	51.2	46.6	55.9	45.4	38.7	52.2	42.0	32.9	51.1	50.9	46.2	55.6

Female, Coloured

	Origin state											
	No disease			One disease			Multimorbidity			Weighted average		
Destination state	Est.	LCI	UCI	Est.	LCI	UCI	Est.	LCI	UCI	Est.	LCI	UCI
No disease	28.6	27.2	30.0	0.0	0.0	0.0	0.0	0.0	0.0	26.9	25.7	28.2
One disease	9.6	8.5	10.7	18.7	16.7	20.7	0.0	0.0	0.0	10.0	8.9	11.2
Multimorbidity	8.7	7.2	10.2	19.1	15.9	22.2	33.2	29.2	37.1	9.3	7.8	10.9
Total	46.9	45.4	48.4	37.7	35.2	40.3	33.2	29.2	37.1	46.3	44.8	47.9

Female, White

	Origin state											
	No disease			One disease			Multimorbidity			Weighted average		
Destination state	Est.	LCI	UCI	Est.	LCI	UCI	Est.	LCI	UCI	Est.	LCI	UCI
No disease	29.9	27.3	32.5	0.0	0.0	0.0	0.0	0.0	0.0	28.2	25.7	30.7
One disease	10.8	8.8	12.8	19.5	16.0	23.1	0.0	0.0	0.0	11.2	9.2	13.3
Multimorbidity	12.7	9.9	15.6	27.7	22.3	33.1	44.4	39.1	49.6	13.7	10.7	16.6
Total	53.5	51.2	55.7	47.3	43.5	51.0	44.4	39.1	49.6	53.1	50.8	55.4

Model 3: Stratified by sex and education

Male, Less than secondary school

	Origin state											
	No disease			One disease			Multimorbidity			Weighted average		
Destination state	Est.	LCI	UCI	Est.	LCI	UCI	Est.	LCI	UCI	Est.	LCI	UCI
No disease	27.5	26.5	28.5	0.0	0.0	0.0	0.0	0.0	0.0	26.5	25.6	27.5
One disease	6.4	5.8	7.0	15.8	14.4	17.2	0.0	0.0	0.0	6.7	6.0	7.3
Multimorbidity	2.6	2.1	3.1	6.4	5.0	7.8	15.2	12.6	17.8	2.8	2.2	3.3
Total	36.5	35.4	37.6	22.2	20.6	23.9	15.2	12.6	17.8	36.0	34.9	37.1

Male, Secondary school

	Origin state											
	No disease			One disease			Multimorbidity			Weighted average		
Destination state	Est.	LCI	UCI	Est.	LCI	UCI	Est.	LCI	UCI	Est.	LCI	UCI
No disease	30.5	29.5	31.6	0.0	0.0	0.0	0.0	0.0	0.0	29.5	28.4	30.5
One disease	6.6	5.9	7.3	16.9	15.5	18.4	0.0	0.0	0.0	6.9	6.2	7.6
Multimorbidity	2.5	1.9	3.1	6.6	5.1	8.1	15.7	13.1	18.2	2.7	2.1	3.3
Total	39.6	38.4	40.8	23.5	21.8	25.2	15.7	13.1	18.2	39.0	37.8	40.3

Male, Post-secondary education

	Origin state											
	No disease			One disease			Multimorbidity			Weighted average		
Destination state	Est.	LCI	UCI	Est.	LCI	UCI	Est.	LCI	UCI	Est.	LCI	UCI
No disease	34.9	33.0	36.7	0.0	0.0	0.0	0.0	0.0	0.0	33.6	31.8	35.4
One disease	8.5	7.2	9.9	20.5	17.8	23.2	0.0	0.0	0.0	8.9	7.5	10.3
Multimorbidity	4.5	3.1	5.8	12.4	8.8	16.0	25.0	19.9	30.2	4.8	3.4	6.2
Total	47.9	45.7	50.0	32.9	29.4	36.4	25.0	19.9	30.2	47.3	45.1	49.5

Female, Less than secondary school

	Origin state											
	No disease			One disease			Multimorbidity			Weighted average		
Destination state	Est.	LCI	UCI	Est.	LCI	UCI	Est.	LCI	UCI	Est.	LCI	UCI
No disease	26.5	25.6	27.5	0.0	0.0	0.0	0.0	0.0	0.0	25	24.1	25.9
One disease	9.6	8.9	10.2	18.4	16.9	19.9	0.0	0.0	0.0	10	9.3	10.7
Multimorbidity	7.5	6.7	8.3	15.7	13.6	17.8	28.6	25.4	31.9	8	7.1	8.9
Total	43.6	42.6	44.6	34.1	32.2	36.0	28.6	25.4	31.9	43	42.0	44.1

Female, Secondary school

	Origin state											
	No disease			One disease			Multimorbidity			Weighted average		
Destination state	Est.	LCI	UCI	Est.	LCI	UCI	Est.	LCI	UCI	Est.	LCI	UCI
No disease	29.4	28.4	30.3	0.0	0.0	0.0	0.0	0.0	0.0	27.7	26.8	28.6
One disease	9.7	8.9	10.6	19.6	18.0	21.2	0.0	0.0	0.0	10.2	9.4	11.1
Multimorbidity	7.0	6.0	8.1	15.8	13.5	18.2	29.3	25.9	32.7	7.6	6.4	8.7
Total	46.1	44.9	47.4	35.4	33.4	37.5	29.3	25.9	32.7	45.5	44.2	46.7

Female, Post-secondary education

	Origin state											
	No disease			One disease			Multimorbidity			Weighted average		
Destination state	Est.	LCI	UCI	Est.	LCI	UCI	Est.	LCI	UCI	Est.	LCI	UCI
No disease	32.4	30.6	34.2	0.0	0.0	0.0	0.0	0.0	0.0	30.5	28.8	32.2
One disease	11.2	9.6	12.8	21.7	18.7	24.7	0.0	0.0	0.0	11.7	10.1	13.4
Multimorbidity	9.8	7.7	11.8	23.3	18.9	27.8	40.4	35.4	45.3	10.6	8.4	12.8
Total	53.3	51.5	55.1	45.0	41.8	48.3	40.4	35.4	45.3	52.8	50.9	54.7

Model 4: Stratified by sex and race*binary education interaction

Male, African, Less than secondary school

	Origin state											
	No disease			One disease			Multimorbidity			Weighted average		
Destination state	Est.	LCI	UCI	Est.	LCI	UCI	Est.	LCI	UCI	Est.	LCI	UCI
No disease	27.6	26.5	28.7	0.0	0.0	0.0	0.0	0.0	0	26.6	25.6	27.7
One disease	6.5	5.8	7.1	16.1	14.6	17.6	0.0	0.0	0	6.8	6.1	7.5
Multimorbidity	2.5	2.0	3.0	6.4	4.9	7.9	15.3	12.7	18	2.7	2.1	3.2
Total	36.6	35.4	37.7	22.5	20.7	24.2	15.3	12.7	18	36.1	34.9	37.2

Male, African, Secondary school or more

	Origin state											
	No disease			One disease			Multimorbidity			Weighted average		
Destination state	Est.	LCI	UCI	Est.	LCI	UCI	Est.	LCI	UCI	Est.	LCI	UCI
No disease	30.9	29.8	31.9	0.0	0.0	0.0	0.0	0.0	0.0	29.8	28.7	30.8
One disease	6.3	5.6	7.0	17.0	15.5	18.4	0.0	0.0	0.0	6.6	5.9	7.4
Multimorbidity	2.0	1.5	2.5	5.6	4.3	6.9	14.3	11.9	16.7	2.1	1.6	2.6
Total	39.1	37.9	40.4	22.5	20.9	24.2	14.3	11.9	16.7	38.5	37.3	39.8

Male, Coloured, Less than secondary school

	Origin state											
	No disease			One disease			Multimorbidity			Weighted average		
Destination state	Est.	LCI	UCI	Est.	LCI	UCI	Est.	LCI	UCI	Est.	LCI	UCI
No disease	27.3	25.6	29.0	0.0	0.0	0.0	0.0	0.0	0.0	26.3	24.7	28.0
One disease	6.2	5.2	7.2	15.2	13.3	17.1	0.0	0.0	0.0	6.5	5.4	7.5
Multimorbidity	2.9	2.1	3.8	7.4	5.3	9.6	16.7	13.3	20.1	3.1	2.2	4.0
Total	36.4	34.5	38.2	22.6	20.3	25.0	16.7	13.3	20.1	35.9	34.0	37.7

Male, Coloured, Secondary school or more

	Origin state											
	No disease			One disease			Multimorbidity			Weighted average		
Destination state	Est.	LCI	UCI	Est.	LCI	UCI	Est.	LCI	UCI	Est.	LCI	UCI
No disease	33.7	31.4	36.0	0.0	0.0	0.0	0.0	0.0	0.0	32.5	30.3	34.8
One disease	8.5	6.8	10.2	20.1	16.8	23.3	0.0	0.0	0.0	8.9	7.1	10.6
Multimorbidity	4.5	2.8	6.2	12.1	7.8	16.5	24.7	18.7	30.6	4.8	3.0	6.5
Total	46.7	43.8	49.5	32.2	27.9	36.5	24.7	18.7	30.6	46.1	43.3	49.0

Male, White, Less than secondary school

	Origin state											
	No disease			One disease			Multimorbidity			Weighted average		
Destination state	Est.	LCI	UCI	Est.	LCI	UCI	Est.	LCI	UCI	Est.	LCI	UCI
No disease	24.8	16.0	33.7	0.0	0.0	0.0	0	0.0	0.0	23.9	15.4	32.5
One disease	10.4	3.8	17.0	18.8	9.4	28.2	0	0.0	0.0	10.6	4.0	17.3
Multimorbidity	8.3	-0.2	16.8	16.5	1.3	31.8	30	11.6	48.5	8.6	-0.1	17.3
Total	43.5	32.6	54.4	35.3	21.1	49.6	30	11.6	48.5	43.2	32.2	54.1

Male, White, Secondary school or more

	Origin state											
	No disease			One disease			Multimorbidity			Weighted average		
Destination state	Est.	LCI	UCI	Est.	LCI	UCI	Est.	LCI	UCI	Est.	LCI	UCI
No disease	32.2	29.4	34.9	0.0	0.0	0.0	0	0.0	0.0	31.0	28.4	33.7
One disease	8.6	6.8	10.4	18.7	15.4	22.1	0	0.0	0.0	8.9	7.0	10.7
Multimorbidity	6.2	4.1	8.3	15.8	10.7	20.9	29	22.6	35.4	6.6	4.4	8.8
Total	46.9	44.0	49.9	34.5	29.9	39.1	29	22.6	35.4	46.5	43.5	49.5

Female, African, Less than secondary school

	Origin state											
	No disease			One disease			Multimorbidity			Weighted average		
Destination state	Est.	LCI	UCI	Est.	LCI	UCI	Est.	LCI	UCI	Est.	LCI	UCI
No disease	26.5	25.5	27.6	0.0	0.0	0.0	0	0.0	0.0	25.0	24.1	26.0
One disease	9.7	9.0	10.5	18.5	16.9	20.2	0	0.0	0.0	10.2	9.4	10.9
Multimorbidity	7.4	6.5	8.3	15.8	13.6	18.0	29	25.7	32.3	8.0	7.0	8.9
Total	43.7	42.6	44.8	34.4	32.4	36.3	29	25.7	32.3	43.2	42.0	44.3

Female, African, Secondary school or more

	Origin state											
	No disease			One disease			Multimorbidity			Weighted average		
Destination state	Est.	LCI	UCI	Est.	LCI	UCI	Est.	LCI	UCI	Est.	LCI	UCI
No disease	29.8	28.9	30.8	0.0	0.0	0.0	0.0	0	0.0	28.1	27.2	29.0
One disease	9.6	8.7	10.5	19.9	18.3	21.5	0.0	0	0.0	10.1	9.2	11.0
Multimorbidity	6.0	5.0	7.1	14.2	11.9	16.5	27.4	24	30.7	6.5	5.4	7.7
Total	45.5	44.2	46.7	34.0	32.0	36.1	27.4	24	30.7	44.8	43.5	46.1

Female, Coloured, Less than secondary school

	Origin state											
	No disease			One disease			Multimorbidity			Weighted average		
Destination state	Est.	LCI	UCI	Est.	LCI	UCI	Est.	LCI	UCI	Est.	LCI	UCI
No disease	26.2	24.5	28.0	0.0	0.0	0.0	0.0	0.0	0.0	24.7	23.1	26.3
One disease	9.1	7.8	10.4	17.2	14.9	19.4	0.0	0.0	0.0	9.5	8.2	10.8
Multimorbidity	8.5	6.7	10.2	17.8	14.2	21.4	30.9	26.5	35.4	9.0	7.2	10.9
Total	43.8	41.9	45.6	35.0	32.1	37.9	30.9	26.5	35.4	43.3	41.4	45.2

Female, Coloured, Secondary school or more

	Origin state											
	No disease			One disease			Multimorbidity			Weighted average		
Destination state	Est.	LCI	UCI	Est.	LCI	UCI	Est.	LCI	UCI	Est.	LCI	UCI
No disease	31.3	29.0	33.6	0.0	0.0	0.0	0.0	0.0	0.0	29.5	27.3	31.6
One disease	11.1	9.1	13.2	21.0	17.4	24.7	0.0	0.0	0.0	11.6	9.5	13.7
Multimorbidity	10.2	7.5	12.8	23.6	18.0	29.1	40.3	34.4	46.2	11.0	8.2	13.8
Total	52.6	50.2	55.0	44.6	40.6	48.7	40.3	34.4	46.2	52.1	49.6	54.6

Female, White, Less than secondary school

	Origin state											
	No disease			One disease			Multimorbidity			Weighted average		
Destination state	Est.	LCI	UCI	Est.	LCI	UCI	Est.	LCI	UCI	Est.	LCI	UCI
No disease	22.2	13.6	30.9	0.0	0.0	0.0	0.0	0.0	0.0	20.9	12.8	29.1
One disease	12.5	5.0	20.0	18.9	8.6	29.2	0.0	0.0	0.0	12.8	5.2	20.4
Multimorbidity	16.6	5.3	27.9	28.8	12.3	45.3	45.2	29.9	60.5	17.4	5.9	28.9
Total	51.3	42.6	60.0	47.7	36.0	59.4	45.2	29.9	60.5	51.1	42.2	60.0

Female, White, Secondary school or more

	Origin state											
	No disease			One disease			Multimorbidity			Weighted average		
Destination state	Est.	LCI	UCI	Est.	LCI	UCI	Est.	LCI	UCI	Est.	LCI	UCI
No disease	29.5	26.7	32.3	0	0.0	0.0	0.0	0.0	0.0	27.8	25.2	30.4
One disease	10.7	8.6	12.9	19	15.3	22.7	0.0	0.0	0.0	11.1	8.9	13.3
Multimorbidity	12.9	9.9	16.0	28	22.3	33.7	44.4	38.9	49.8	13.9	10.7	17.1
Total	53.2	50.8	55.5	47	43.1	50.9	44.4	38.9	49.8	52.8	50.4	55.3

4 Estimate life expectancy from age 40

4.1 Sample characteristics

	Male (n=3,266)	Female (n=5,404)	Overall (n=8,670)
**Age (years)**			
Mean (SD)	55.3 (11.0)	56.3 (11.9)	56.0 (11.6)
Median [Min, Max]	53.4 [40.0, 99.2]	54.1 [40.0, 106]	53.8 [40.0, 106]
**Race**			
African	2,406 (73.7%)	4,268 (79.0%)	6,674 (77.0%)
Asian/Indian	53 (1.6%)	74 (1.4%)	127 (1.5%)
Coloured	563 (17.2%)	772 (14.3%)	1,335 (15.4%)
White	244 (7.5%)	290 (5.4%)	534 (6.2%)
**Education level**			
Less than secondary school	2,220 (68.0%)	3,973 (73.5%)	6,193 (71.4%)
Secondary school	703 (21.5%)	980 (18.1%)	1,683 (19.4%)
Post-secondary education	343 (10.5%)	451 (8.3%)	794 (9.2%)
**Residence**			
Rural	1,522 (46.6%)	2,898 (53.6%)	4,420 (51.0%)
Urban	1,744 (53.4%)	2,506 (46.4%)	4,250 (49.0%)
**Origin 'from' state**			
No disease	1,998 (61.2%)	2,579 (47.7%)	4,577 (52.8%)
One disease	933 (28.6%)	1,939 (35.9%)	2,872 (33.2%)
Multimorbidity	335 (10.3%)	886 (16.4%)	1,221 (14.1%)

4.2 Expectancy estimates

Model 1: Stratified by sex

Male

	Origin state											
	No disease			One disease			Multimorbidity			Weighted average		
Destination state	Est.	LCI	UCI	Est.	LCI	UCI	Est.	LCI	UCI	Est.	LCI	UCI
No disease	11.2	10.6	11.7	0.0	0.0	0.0	0.0	0.0	0.0	7.6	7.2	7.9
One disease	7.5	7.1	8.0	11.2	10.6	11.9	0.0	0.0	0.0	8.0	7.5	8.5
Multimorbidity	7.8	7.2	8.5	11.8	10.9	12.8	19.9	18.7	21.2	9.7	8.9	10.4
Total	26.6	25.8	27.3	23.1	22.1	24.0	19.9	18.7	21.2	25.2	24.4	26.0

Female

	Origin state											
	No disease			One disease			Multimorbidity			Weighted average		
Destination state	Est.	LCI	UCI	Est.	LCI	UCI	Est.	LCI	UCI	Est.	LCI	UCI
No disease	10.9	10.4	11.4	0.0	0.0	0.0	0.0	0.0	0.0	6.5	6.2	6.8
One disease	8.9	8.5	9.3	12.2	11.6	12.8	0.0	0.0	0.0	8.9	8.5	9.3
Multimorbidity	12.3	11.7	13.0	17.6	16.7	18.4	27.4	26.3	28.5	15.5	14.8	16.2
Total	32.2	31.5	32.8	29.8	29.0	30.6	27.4	26.3	28.5	30.9	30.3	31.6

Model 2: Stratified by sex and race

Male, African

	Origin state											
	No disease			One disease			Multimorbidity			Weighted average		
Destination state	Est.	LCI	UCI	Est.	LCI	UCI	Est.	LCI	UCI	Est.	LCI	UCI
No disease	11.3	10.7	11.9	0.0	0.0	0.0	0.0	0.0	0.0	7.6	7.2	8.0
One disease	7.5	7.0	8.0	11.2	10.5	12.0	0.0	0.0	0.0	7.9	7.4	8.5
Multimorbidity	7.5	6.8	8.2	11.4	10.4	12.4	19.4	18.1	20.7	9.3	8.5	10.1
Total	26.2	25.5	27.0	22.6	21.7	23.6	19.4	18.1	20.7	24.9	24.0	25.7

Male, Asian/Indian

	Origin state											
	No disease			One disease			Multimorbidity			Weighted average		
Destination state	Est.	LCI	UCI	Est.	LCI	UCI	Est.	LCI	UCI	Est.	LCI	UCI
No disease	15.3	11.3	19.2	0.0	0.0	0.0	0.0	0.0	0.0	10.3	7.7	13.0
One disease	5.0	3.0	6.9	8.4	5.9	10.9	0.0	0.0	0.0	5.5	3.6	7.4
Multimorbidity	9.5	5.7	13.3	16.6	11.2	21.9	23.9	18.3	29.5	12.3	8.1	16.5
Total	29.7	25.4	34.1	25.0	19.8	30.1	23.9	18.3	29.5	28.1	23.6	32.6

Male, Coloured

	Origin state											
	No disease			One disease			Multimorbidity			Weighted average		
Destination state	Est.	LCI	UCI	Est.	LCI	UCI	Est.	LCI	UCI	Est.	LCI	UCI
No disease	10.0	9.1	10.8	0.0	0.0	0.0	0.0	0.0	0.0	6.7	6.2	7.3
One disease	7.5	6.7	8.3	10.9	9.9	11.9	0.0	0.0	0.0	7.9	7.1	8.6
Multimorbidity	8.1	7.0	9.2	11.7	10.3	13.2	19.7	17.9	21.4	9.8	8.6	11.0
Total	25.5	24.3	26.8	22.6	21.2	24.0	19.7	17.9	21.4	24.4	23.1	25.7

Male, White

	Origin state											
	No disease			One disease			Multimorbidity			Weighted average		
Destination state	Est.	LCI	UCI	Est.	LCI	UCI	Est.	LCI	UCI	Est.	LCI	UCI
No disease	13.4	11.7	15.1	0.0	0.0	0.0	0.0	0.0	0.0	9.0	7.9	10.2
One disease	8.7	7.3	10.0	12.7	10.9	14.5	0.0	0.0	0.0	9.1	7.8	10.5
Multimorbidity	10.1	8.1	12.1	15.9	13.1	18.6	25.7	22.6	28.8	12.6	10.4	14.9
Total	32.1	30.1	34.2	28.6	26.1	31.1	25.7	22.6	28.8	30.8	28.6	33.0

Female, African

	Origin state											
	No disease			One disease			Multimorbidity			Weighted average		
Destination state	Est.	LCI	UCI	Est.	LCI	UCI	Est.	LCI	UCI	Est.	LCI	UCI
No disease	11.0	10.4	11.5	0.0	0.0	0.0	0.0	0.0	0.0	6.6	6.2	6.9
One disease	8.9	8.5	9.4	12.3	11.6	12.9	0.0	0.0	0.0	8.9	8.5	9.4
Multimorbidity	12.1	11.4	12.7	17.2	16.3	18.2	27.0	25.9	28.2	15.2	14.4	16.0
Total	32.0	31.3	32.6	29.5	28.7	30.3	27.0	25.9	28.2	30.7	30.0	31.4

Female, Asian/Indian

	Origin state											
	No disease			One disease			Multimorbidity			Weighted average		
Destination state	Est.	LCI	UCI	Est.	LCI	UCI	Est.	LCI	UCI	Est.	LCI	UCI
No disease	15.0	11.0	19.0	0.0	0.0	0.0	0.0	0.0	0.0	9.0	6.6	11.4
One disease	5.8	3.6	7.9	8.9	6.1	11.6	0.0	0.0	0.0	6.1	4.0	8.1
Multimorbidity	14.1	10.0	18.3	23.0	17.9	28.2	31.2	26.4	36.0	18.6	14.3	22.9
Total	34.9	31.4	38.4	31.9	27.5	36.3	31.2	26.4	36.0	33.6	29.8	37.5

Female, Coloured

	Origin state											
	No disease			One disease			Multimorbidity			Weighted average		
Destination state	Est.	LCI	UCI	Est.	LCI	UCI	Est.	LCI	UCI	Est.	LCI	UCI
No disease	9.7	8.8	10.5	0.0	0.0	0.0	0.0	0.0	0.0	5.8	5.3	6.3
One disease	8.9	8.0	9.7	11.9	10.9	12.9	0.0	0.0	0.0	8.8	8.0	9.6
Multimorbidity	13.0	11.7	14.3	17.7	16.1	19.3	27.3	25.6	29.0	15.9	14.5	17.3
Total	31.5	30.3	32.7	29.6	28.2	30.9	27.3	25.6	29.0	30.5	29.2	31.7

Female, White

	Origin state											
	No disease			One disease			Multimorbidity			Weighted average		
Destination state	Est.	LCI	UCI	Est.	LCI	UCI	Est.	LCI	UCI	Est.	LCI	UCI
No disease	12.8	11.1	14.4	0.0	0.0	0.0	0.0	0.0	0.0	7.7	6.7	8.6
One disease	9.8	8.3	11.2	13.4	11.5	15.3	0.0	0.0	0.0	9.8	8.3	11.2
Multimorbidity	14.2	12.1	16.3	21.2	18.5	23.9	32.7	30.2	35.2	18.3	16.0	20.5
Total	36.8	35.2	38.3	34.6	32.6	36.6	32.7	30.2	35.2	35.7	33.9	37.5

Model 3: Stratified by sex and education

Male, Less than secondary school

	Origin state											
	No disease			One disease			Multimorbidity			Weighted average		
Destination state	Est.	LCI	UCI	Est.	LCI	UCI	Est.	LCI	UCI	Est.	LCI	UCI
No disease	10.6	10.0	11.3	0.0	0.0	0.0	0	0.0	0.0	7.2	6.8	7.6
One disease	7.3	6.8	7.8	10.8	10.1	11.5	0	0.0	0.0	7.7	7.2	8.2
Multimorbidity	7.6	6.9	8.3	11.3	10.3	12.3	19	17.7	20.4	9.3	8.5	10.1
Total	25.5	24.7	26.3	22.1	21.1	23.1	19	17.7	20.4	24.2	23.3	25.0

Male, Secondary school

	Origin state											
	No disease			One disease			Multimorbidity			Weighted average		
Destination state	Est.	LCI	UCI	Est.	LCI	UCI	Est.	LCI	UCI	Est.	LCI	UCI
No disease	11.5	10.7	12.4	0.0	0.0	0.0	0.0	0.0	0.0	7.8	7.2	8.4
One disease	7.8	7.0	8.6	11.4	10.4	12.4	0.0	0.0	0.0	8.2	7.4	9.0
Multimorbidity	8.7	7.5	9.9	13.1	11.4	14.7	21.5	19.5	23.4	10.7	9.3	12.0
Total	28.0	26.6	29.4	24.5	22.9	26.1	21.5	19.5	23.4	26.6	25.2	28.1

Male, Post-secondary education

	Origin state											
	No disease			One disease			Multimorbidity			Weighted average		
Destination state	Est.	LCI	UCI	Est.	LCI	UCI	Est.	LCI	UCI	Est.	LCI	UCI
No disease	13.6	12.2	15.0	0.0	0.0	0.0	0.0	0.0	0.0	9.2	8.2	10.1
One disease	9.5	8.2	10.8	13.9	12.2	15.6	0.0	0.0	0.0	10.0	8.7	11.3
Multimorbidity	9.0	7.2	10.8	14.3	11.7	16.9	24.4	21.3	27.5	11.4	9.4	13.5
Total	32.1	30.1	34.1	28.2	25.7	30.7	24.4	21.3	27.5	30.6	28.4	32.7

Female, Less than secondary school

	Origin state											
	No disease			One disease			Multimorbidity			Weighted average		
Destination state	Est.	LCI	UCI	Est.	LCI	UCI	Est.	LCI	UCI	Est.	LCI	UCI
No disease	10.4	9.9	11.0	0.0	0.0	0.0	0.0	0.0	0.0	6.2	5.9	6.6
One disease	8.7	8.3	9.2	11.9	11.2	12.6	0.0	0.0	0.0	8.7	8.2	9.2
Multimorbidity	12.3	11.6	13.0	17.2	16.3	18.2	26.7	25.5	27.9	15.3	14.5	16.1
Total	31.4	30.8	32.1	29.1	28.2	29.9	26.7	25.5	27.9	30.2	29.5	31.0

Female, Secondary school

	Origin state											
	No disease			One disease			Multimorbidity			Weighted average		
Destination state	Est.	LCI	UCI	Est.	LCI	UCI	Est.	LCI	UCI	Est.	LCI	UCI
No disease	11.2	10.4	12.0	0.0	0.0	0.0	0.0	0.0	0.0	6.7	6.2	7.2
One disease	9.1	8.3	9.9	12.3	11.3	13.4	0.0	0.0	0.0	9.1	8.3	9.9
Multimorbidity	13.3	12.0	14.6	19.0	17.3	20.6	29.1	27.3	30.9	16.7	15.2	18.1
Total	33.6	32.4	34.8	31.3	29.9	32.7	29.1	27.3	30.9	32.4	31.1	33.7

Female, Post-secondary education

	Origin state											
	No disease			One disease			Multimorbidity			Weighted average		
Destination state	Est.	LCI	UCI	Est.	LCI	UCI	Est.	LCI	UCI	Est.	LCI	UCI
No disease	13.0	11.7	14.4	0.0	0.0	0.0	0.0	0.0	0.0	7.8	7.0	8.6
One disease	10.8	9.4	12.2	14.8	13.0	16.6	0.0	0.0	0.0	10.8	9.4	12.2
Multimorbidity	12.8	10.9	14.7	19.4	16.9	22.0	31.6	29.0	34.3	16.8	14.7	18.9
Total	36.7	35.1	38.2	34.3	32.3	36.3	31.6	29.0	34.3	35.4	33.6	37.2

Model 4: Stratified by sex and race*binary education interaction

Male, African, Less than secondary school

	Origin state											
	No disease			One disease			Multimorbidity			Weighted average		
Destination state	Est.	LCI	UCI	Est.	LCI	UCI	Est.	LCI	UCI	Est.	LCI	UCI
No disease	11.0	10.3	11.6	0.0	0.0	0.0	0.0	0.0	0.0	7.4	7.0	7.8
One disease	7.3	6.8	7.9	11.0	10.2	11.7	0.0	0.0	0.0	7.8	7.2	8.3
Multimorbidity	7.5	6.7	8.2	11.2	10.2	12.3	19.1	17.7	20.5	9.2	8.4	10.1
Total	25.8	24.9	26.6	22.2	21.2	23.3	19.1	17.7	20.5	24.4	23.5	25.3

Male, African, Secondary school or more

	Origin state											
	No disease			One disease			Multimorbidity			Weighted average		
Destination state	Est.	LCI	UCI	Est.	LCI	UCI	Est.	LCI	UCI	Est.	LCI	UCI
No disease	11.9	10.9	12.8	0.0	0.0	0.0	0.0	0.0	0.0	8.0	7.4	8.6
One disease	8.0	7.1	8.9	11.9	10.8	13.0	0.0	0.0	0.0	8.4	7.6	9.3
Multimorbidity	7.8	6.6	9.1	12.0	10.3	13.7	20.5	18.4	22.5	9.8	8.4	11.2
Total	27.7	26.2	29.2	23.9	22.2	25.6	20.5	18.4	22.5	26.2	24.7	27.8

Male, Coloured, Less than secondary school

	Origin state											
	No disease			One disease			Multimorbidity			Weighted average		
Destination state	Est.	LCI	UCI	Est.	LCI	UCI	Est.	LCI	UCI	Est.	LCI	UCI
No disease	9.3	8.4	10.3	0.0	0.0	0.0	0.0	0.0	0.0	6.3	5.7	6.9
One disease	7.3	6.4	8.1	10.6	9.5	11.6	0.0	0.0	0.0	7.6	6.8	8.5
Multimorbidity	7.8	6.6	9.0	11.1	9.5	12.7	18.7	16.9	20.6	9.4	8.1	10.7
Total	24.4	23.0	25.8	21.7	20.1	23.2	18.7	16.9	20.6	23.3	21.9	24.7

Male, Coloured, Secondary school or more

	Origin state											
	No disease			One disease			Multimorbidity			Weighted average		
Destination state	Est.	LCI	UCI	Est.	LCI	UCI	Est.	LCI	UCI	Est.	LCI	UCI
No disease	11.2	9.6	12.8	0.0	0.0	0.0	0	0.0	0.0	7.6	6.5	8.6
One disease	8.2	6.6	9.8	11.7	9.8	13.6	0	0.0	0.0	8.5	7.0	10.1
Multimorbidity	9.7	7.2	12.1	14.2	10.9	17.5	23	19.3	26.8	11.7	9.0	14.5
Total	29.0	26.3	31.8	25.9	22.7	29.1	23	19.3	26.8	27.8	24.9	30.8

Male, White, Less than secondary school

	Origin state											
	No disease			One disease			Multimorbidity			Weighted average		
Destination state	Est.	LCI	UCI	Est.	LCI	UCI	Est.	LCI	UCI	Est.	LCI	UCI
No disease	10.8	4.8	16.9	0.0	0.0	0.0	0.0	0.0	0.0	7.3	3.2	11.4
One disease	4.9	2.0	7.7	7.2	3.8	10.6	0.0	0.0	0.0	5.1	2.3	7.9
Multimorbidity	15.9	7.7	24.1	22.6	13.4	31.8	29.6	20.6	38.5	18.5	10.2	26.9
Total	31.6	24.1	39.1	29.8	21.3	38.3	29.6	20.6	38.5	31.0	23.3	38.7

Male, White, Secondary school or more

	Origin state											
	No disease			One disease			Multimorbidity			Weighted average		
Destination state	Est.	LCI	UCI	Est.	LCI	UCI	Est.	LCI	UCI	Est.	LCI	UCI
No disease	13.4	11.6	15.1	0.0	0.0	0.0	0	0.0	0.0	9.0	7.9	10.2
One disease	9.0	7.5	10.5	13.2	11.3	15.1	0	0.0	0.0	9.5	8.0	10.9
Multimorbidity	9.6	7.6	11.7	15.1	12.3	18.0	25	21.8	28.3	12.1	9.8	14.4
Total	32.0	29.9	34.2	28.3	25.7	30.9	25	21.8	28.3	30.6	28.3	32.9

Female, African, Less than secondary school

	Origin state											
	No disease			One disease			Multimorbidity			Weighted average		
Destination state	Est.	LCI	UCI	Est.	LCI	UCI	Est.	LCI	UCI	Est.	LCI	UCI
No disease	10.7	10.1	11.3	0.0	0.0	0.0	0.0	0.0	0	6.4	6.0	6.8
One disease	8.8	8.3	9.3	12.1	11.3	12.8	0.0	0.0	0	8.8	8.3	9.3
Multimorbidity	12.1	11.4	12.9	17.1	16.1	18.2	26.8	25.5	28	15.2	14.3	16.0
Total	31.6	30.9	32.3	29.2	28.3	30.1	26.8	25.5	28	30.4	29.6	31.2

Female, African, Secondary school or more

	Origin state											
	No disease			One disease			Multimorbidity			Weighted average		
Destination state	Est.	LCI	UCI	Est.	LCI	UCI	Est.	LCI	UCI	Est.	LCI	UCI
No disease	11.5	10.6	12.4	0.0	0.0	0.0	0.0	0.0	0.0	6.9	6.4	7.4
One disease	9.4	8.5	10.3	12.9	11.7	14.1	0.0	0.0	0.0	9.4	8.5	10.3
Multimorbidity	12.3	10.9	13.7	17.8	16.0	19.6	28.1	26.1	30.1	15.6	14.1	17.2
Total	33.2	31.9	34.6	30.7	29.1	32.3	28.1	26.1	30.1	31.9	30.5	33.4

Female, Coloured, Less than secondary school

	Origin state											
	No disease			One disease			Multimorbidity			Weighted average		
Destination state	Est.	LCI	UCI	Est.	LCI	UCI	Est.	LCI	UCI	Est.	LCI	UCI
No disease	9.1	8.2	10.1	0.0	0.0	0.0	0.0	0.0	0.0	5.4	4.9	6.0
One disease	8.7	7.8	9.7	11.6	10.5	12.8	0.0	0.0	0.0	8.6	7.7	9.5
Multimorbidity	12.8	11.3	14.2	17.1	15.3	18.9	26.4	24.5	28.3	15.5	14.0	17.1
Total	30.6	29.2	31.9	28.7	27.2	30.2	26.4	24.5	28.3	29.6	28.2	31.0

Female, Coloured, Secondary school or more

	Origin state											
	No disease			One disease			Multimorbidity			Weighted average		
Destination state	Est.	LCI	UCI	Est.	LCI	UCI	Est.	LCI	UCI	Est.	LCI	UCI
No disease	10.8	9.2	12.3	0.0	0.0	0.0	0.0	0.0	0.0	6.4	5.5	7.4
One disease	9.4	7.7	11.1	12.5	10.5	14.6	0.0	0.0	0.0	9.3	7.7	10.9
Multimorbidity	14.3	11.7	17.0	20.0	16.7	23.3	30.5	27.2	33.8	17.8	14.9	20.6
Total	34.5	32.2	36.8	32.6	29.8	35.3	30.5	27.2	33.8	33.5	31.0	36.0

Female, White, Less than secondary school

	Origin state											
	No disease			One disease			Multimorbidity			Weighted average		
Destination state	Est.	LCI	UCI	Est.	LCI	UCI	Est.	LCI	UCI	Est.	LCI	UCI
No disease	10.4	4.4	16.4	0.0	0.0	0.0	0.0	0.0	0	6.2	2.7	9.8
One disease	5.4	2.2	8.5	7.4	3.8	11.0	0.0	0.0	0	5.4	2.5	8.3
Multimorbidity	21.0	13.4	28.6	28.4	21.0	35.8	35.7	29.3	42	24.8	17.7	31.8
Total	36.8	31.4	42.1	35.8	29.8	41.9	35.7	29.3	42	36.4	30.8	42.0

Female, White, Secondary school or more

	Origin state											
	No disease			One disease			Multimorbidity			Weighted average		
Destination state	Est.	LCI	UCI	Est.	LCI	UCI	Est.	LCI	UCI	Est.	LCI	UCI
No disease	12.8	11.1	14.5	0.0	0.0	0.0	0.0	0.0	0.0	7.7	6.7	8.7
One disease	10.2	8.6	11.8	13.9	11.9	16.0	0.0	0.0	0.0	10.2	8.7	11.7
Multimorbidity	13.7	11.5	15.8	20.5	17.7	23.3	32.2	29.5	34.9	17.7	15.3	20.0
Total	36.7	35.0	38.3	34.4	32.3	36.5	32.2	29.5	34.9	35.5	33.7	37.4
